# Active metasurface designs for lensless and detector-limited imaging

**DOI:** 10.1515/nanoph-2024-0704

**Published:** 2025-05-05

**Authors:** Julie Belleville, Prachi Thureja, Harry A. Atwater

**Affiliations:** 6469 Thomas J. Watson Laboratories of Applied Physics, California Institute of Technology, Pasadena, CA 91125, USA

**Keywords:** optical imaging, array factor, active metasurfaces, lensless imaging, single-pixel imaging

## Abstract

The emergence of metasurfaces has enabled lightweight, compact imaging with degrees of freedom which previously required complex optical setups to achieve, such as polarization, wave vector, and spectrum. To date, most metasurface-enabled imaging systems have thus far been ‘passive’, and therefore subject to fundamental information and thickness limits set by the coupling of light to their sensor arrays. We discuss the use of active metasurfaces in low form-factor and low pixel-count imaging systems and introduce a prototypical lensless imaging system concept which employs an active metasurface as a high-frequency, continuously tunable amplitude and phase modulation aperture, coupled to a discrete single-pixel detector. We analyze the scalability of such a platform and computationally demonstrate that a scalable ‘perimeter-control’ addressing architecture – in which a *M* × *N* rectangular array of scattering elements is addressed by only *M* + *N* voltages – is sufficient for image collection, even when scatterers exhibit limited $\left(272^{◦}\right)$ phase control, and undesired amplitude variations. We also address fundamental limits in information collection, image aberrations, and signal-to-noise ratio, highlighting key advantages, limitations, and trade-offs for active metasurface imaging. We generalize our discussion to other active metasurface-enabled imaging configurations and applications. Finally, we consider promising active metasurface material platforms with an outlook towards new directions to enable high-efficiency imaging.

## 1 Introduction

The past 15 years have seen the emergence of three novel imaging concepts: lensless imaging, which enables imaging with near-flat devices [1]; single-pixel imaging, which allows image information to be recovered in applications where only single-pixel (single-detector) sensors are available [2]; and metasurface-based imaging, which has enabled compact imaging in a range of applications including spectral imaging, polarization imaging, depth-of-field sensing, and more [3], [4], [5]. This perspective reflects on these new imaging technologies, addressing the potential for active metasurfaces in future imaging technologies.

In this work, we first review the state-of-the-art for lensless, single-pixel, and metasurface-enabled imaging, introducing key works to which an active metasurface-based imager can be compared. Next, we propose an approach to single-pixel, lensless active metasurface imaging and highlight key advantages that such a platform would have. We then discuss the fabrication of large-scale apertures for such an imager, focusing on different ways to electrically address metasurface scatterers, and numerically demonstrate the viability of a scalable architecture which requires no memory (capacitive) elements and uses only *M* + *N* addressing voltages for a *M* × *N* rectangular array of scatterers. With the viability of the proposed device established, we analyze the key imaging metrics of resolution, field of view (FOV), aberrations, signal-to-noise ratio (SNR) and acquisition times for the device. We compare these results to existing technology and discuss necessary improvements in the state-of-the-art of active metasurfaces. We also briefly discuss other modalities of imaging which active metasurfaces might enable. Finally, using these results, we analyze benefits and drawbacks of various active metasurface platforms in imaging and discuss possible applications of the technology.

## 2 Review of lensless, single-pixel, and metasurface-enabled imaging

**Figure 1:**
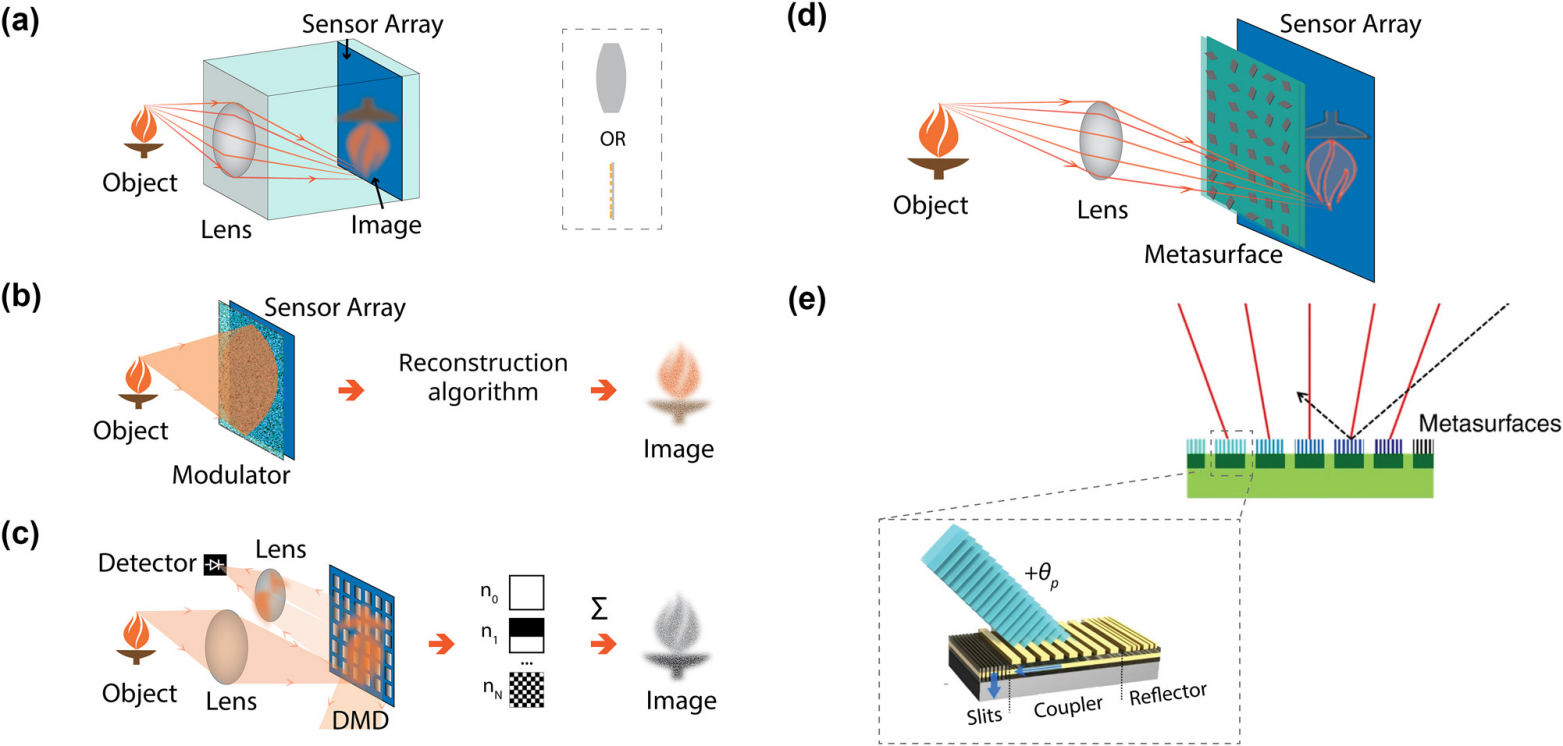
Imaging systems: (a) a standard lens-coupled sensor array imaging system. Light is focused onto the image plane through a lens and collected at each point by a sensor array. Metalenses may be used in place of refractive lenses in such systems. (b) A lensless imaging system. Information from the scene is encoded onto a sensor array through a modulator placed directly against the array. The information collected by the sensor array must be computationally decoded to produce an image. (c) Single-pixel imaging system, using a digital micromirror device (DMD) as an amplitude modulator. The DMD is placed at the image plane and selectively directs light from each pixel either toward or away from a single detector. (d) Metasurface-enabled imaging system. A metasurface is placed in the optical path to enable imaging of unusual degrees of freedom (here: wave vector for edge detection). (e) Lensless, compound-eye imager based on angle-sensitive metasurfaces. Modified with permission from ref. [23]. Creative Commons CC BY.

The performance of conventional imaging systems is dictated by trade-offs between device size, resolution, and field of view (FOV). For example, a pinhole camera uses a fixed sensor array aperture size and a fixed distance between the object and the image detector. The captured image resolution can only be increased by increasing the distance between the pinhole and detector, which in turn decreases the FOV. The diffraction-limited resolution of conventional lens-coupled detector arrays (Figure 1(a)) can be improved by increasing the numerical aperture, but this improvement is often linked to increased system complexity or aberrations at wide FOV [6]. In such systems which passively collect light, the sensor array imposes a critical limit on the number of resolvable points. At the Nyquist limit, a passive system with *N* pixels along a sensor axis can resolve at most *N*/2 points along a given axis. For a specific sensor array, this leads to a fundamental trade-off between resolution and FOV, regardless of how light is acquired.

Further, lens-coupled image sensors require a minimum optical ‘thickness’, related to the geometrical configuration of the information channels in the system [7]. This optical thickness requirement in turn directly dictates how present-day ‘thick’ optical systems are designed and manufactured, and for which optical component assembly costs often dominate the total optical system cost. This has led to an increased interest in lensless imagers (Figure 1(b)) in which an optical encoder is placed in close proximity to a sensor to enable computational reconstruction [1], [8]. These lensless cameras allow for significantly smaller device weight and footprints by eliminating the need for refractive lenses and their focal lengths, making them particularly suited for wearable technologies and other applications where a flat profile or miniaturized form factor is desired. The technology has gained popularity; in 2016, Hitachi demonstrated a lensless camera designed with permeable films and a Fourier transform based reconstruction algorithm [8]. The encoder can be active or passive – with active encoders such as DMDs, single-pixel lensless imaging is also possible [1].

The concept of single-pixel (single-detector) imaging has emerged in the last decade to address the dependence of conventional imaging on sensor array size [2]. While visible light imaging has access to megapixel-scale CMOS image sensor arrays, imaging systems at other wavelengths typically suffer from more limited resolution and higher cost and complexity due to immature image sensor array technology. The cost, size, and operating temperatures of detectors hinders many applications such as cancer diagnosis, artwork inspection, and semiconductor wafer inspection [9]. Single-pixel imaging uses an actively modulated aperture to collect the light scattered by the scene and incident on a detector. This allows the system to utilize the often superior performance (SNR, dynamic range, bandwidth) of single-pixel detectors while preserving spatial resolution, at the cost of longer acquisition times [10]. An exemplary configuration is illustrated in Figure 1(c). A digital micromirror device (DMD) is placed in the image plane of a lens and acts as a binary (on/off) amplitude modulator which directs light at each micromirror either towards or away from a single detector. By displaying orthogonal basis elements *O*_*t*_ across the DMD at different times *t*, an image can be recovered by summing the elements *O*_*t*_ weighted by the measurement at time *t*. In some cases, a compressed sensing basis may replace the orthogonal basis, significantly reducing the number of measurements required to reconstruct a scene [11].

In contrast to lensless and single-pixel imaging, metasurface-enabled imaging did not emerge to address a particular challenge of conventional imaging, but rather a range of objectives including reducing the weight/bulk of systems [12], [13], [14]; imaging unconventional degrees of freedom such as polarization [15], [16], wave vector [17], [18], and spectrum [19], [20]; enabling super-resolution imaging [21], [22]; and achieving wide FOV imaging [23], [24]. Metasurfaces are planar arrays of subwavelength scatterers which allow unprecedented and compact control of degrees of freedom in light including amplitude, phase and polarization [25]. To date, most reports about imaging have used passive metasurfaces, where the subwavelength scatterers have optical properties that are fixed at the time of fabrication. In contrast, the refractive index of active metasurface scatterers can be dynamically tuned by external stimuli such as an applied voltage, allowing reconfigurable control over the wavefronts of light. Active metasurface scattering elements can be tuned through a variety of methods such as mechanical actuation [26], liquid crystal reorientation [27], carrier accumulation [28], and the electro-optic effect [29] – each offering different trade-offs in terms of technological maturity, operation wavelength, operation mode (reflection or transmission), and modulation speeds [25]. With proper choice of material platform, active metasurfaces have the potential to achieve highly desirable characteristics for imaging such as high modulation speeds (>10 MHz) to reduce image acquisition time, compact size, wide FOV, and continuous-valued phase and amplitude modulation [30]. In contrast, DMDs are limited to binary amplitude modulation at ∼30 kHz and must be used jointly with appropriate lenses to achieve wide FOVs [31].

Metasurface-enabled imaging can broadly be categorized into four classes. First, tremendous effort has gone into the development of metalenses [32], which replace bulk refractive lenses by lighter, flat optics. Metalenses are metasurfaces in which the scattering elements are designed to apply spatially varying phase profiles for focusing. While early metalenses suffered from low efficiencies due to their plasmonic nature [33], huge strides have been made in the development of high-efficiency, wide FOV, and achromatic metalenses [34], [35], [36]. Metalenses have also been used to independently control distinct degrees of freedom of light, such as polarization and wavelength [37], [38], [39], concurrently with computational reconstruction for depth sensing through depth-dependent point spread functions (PSFs) [40], and in light-field imaging [18]. They have been used in fields ranging from microscopy to astronomy [14], [41], and have greatly benefited from the availability of computational post-processing [5]. Despite their successes, however, metalens-based imaging systems remain subject to the same fundamental trade-offs between system size, FOV, resolution, and detector pixel density as other lens-coupled sensor arrays (Figure 1(a)).

Second, metasurfaces have been introduced as additional elements in otherwise standard optical imaging systems (Figure 1(d)), once again facilitating the acquisition of different degrees of freedom of light. Demonstrations achieved by such systems include image edge-detection by the control of transmitted wave vectors [17] and full-Stokes polarization imaging [15], [16]. Additionally, metasurfaces have enabled novel sensor designs in hyperspectral imaging [42], terahertz (THz) sensing [43], and fast polarization sensing [44]. Once again, the limitations of lens-coupled sensor arrays remain.

Third, metasurfaces have been used as part of projection or light-focusing mechanisms for sensing, such as in beam deflection for LiDAR applications [45], or to create high-resolution patterns across imaging targets for super-resolution imaging [22]. While very valuable, these approaches require interaction of the imaging system with the object being imaged via active illumination, which can be either unfeasible (when the object is far away) or undesirable (when the imaging process should not influence the object).

Finally, metasurfaces have been used in lensless imaging applications, enabling wide FOV information collection. A common approach to lensless imaging is to use encoding optics to encode object information into a sensor array, and to subsequently computationally decode the information [1]; it has been proposed that metasurfaces be used as such coding aperture, due to their unique control over properties of wavefronts [46]. In Figure 1(e), we highlight a contrasting approach from the work of Kogos et al. on a lensless, compound-eye imager based on angle-sensitive metasurface elements [23]. In this work, the full imager is composed of multiple different pixels, each capable of coupling light from a given polar angle into a photoactive material, while rejecting light coming from other directions. This allows images to be reconstructed across a very wide FOV (±75°) with relatively simple computational reconstruction; the static nature of the device, however, requires the splitting of the aperture into different angular pixels. The angular full width half max (FWHM) of each pixel is therefore limited by the pixel size within the full aperture.

Some efforts have been made to include active elements in metasurface-enabled imaging. For example, tunable focal lengths have been demonstrated in metalenses [26], [28]. However, there remains a gap in the literature when it comes to leveraging advantages of metasurfaces – control over varied degrees of freedom (polarization, spectrum, phase), small form factors, and wide FOVs – in detector-limited applications, where active reconfiguration is the main mechanism by which object information can be resolved. It is this gap which we target in the subsequent discussion.

## 3 A lensless active metasurface imager, for detector-limited applications

**Figure 2:**
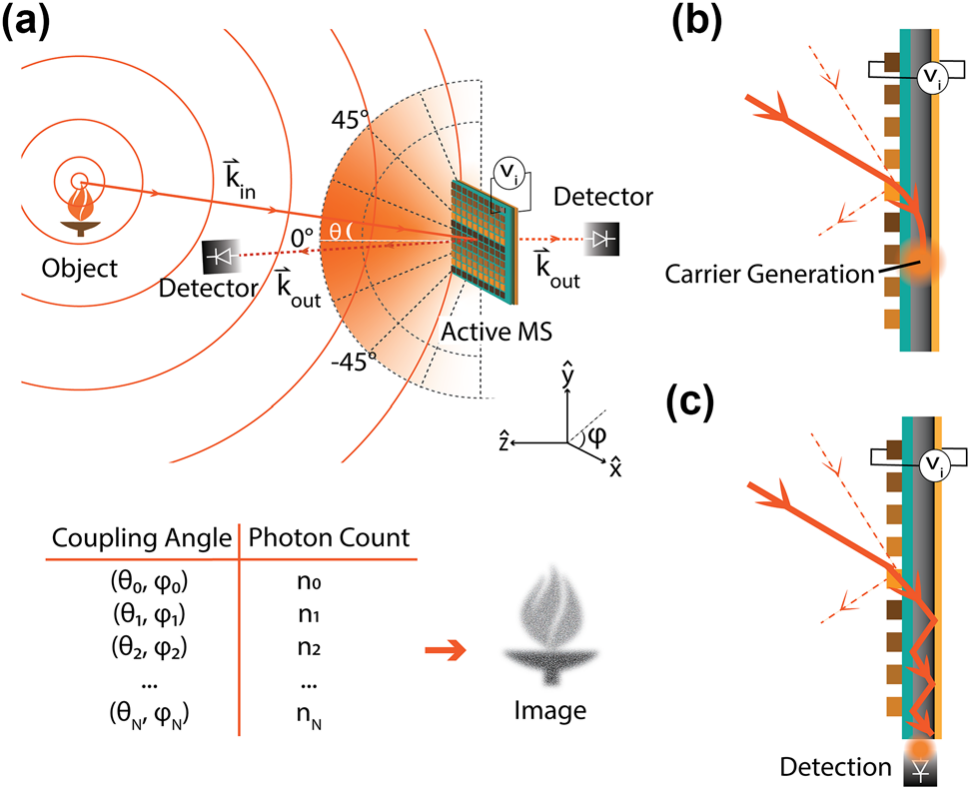
Proposed active metasurface imager: (a) proposed active metasurface single-pixel lensless imaging device. Light is incident from the far field onto an active metasurface, which selectively couples inbound light as a function of wavevectors ${\vec{k}}_{in}$ to a single-pixel detector, schematically represented at the outbound wavevector ${\vec{k}}_{\text{out}}$ . The coupling can be dynamically modified via applied stimulus (depicted here as a voltage) on each metasurface scatterer. (b) Active angular selection of the light coupling into an active material leads to carrier generation, enabling on-chip detection. (c) Active angular selection of the light coupling into a waveguide allows detection at an edge-coupled, external detector element.

To study the value of active metasurfaces in imaging, this section introduces a lensless imaging system composed of an active metasurface and single-pixel detector, which later sections will compare to existing imaging systems. This active metasurface-based single-pixel imaging device architecture is conceptually described in Figure 2(a). The active metasurface element selectively couples light from the far field into a single detector element, replacing both the lens and modulator elements most commonly present in single-pixel imaging setups. The imaging approach is similar to that presented in Kogos et al.’s work (Figure 1(e)) [23], but with a very major distinction: rather than relying on static, *spatially-separated* grating couplers to isolate light from different angles, we propose to collect light from each angle across the full aperture in actively reconfigured, *time-separated* measurements. While this approach allows for much greater resolution, it increases system complexity, as detailed in the following.

In this system, the coupling between different incident wave vectors and the detector is controlled through the voltage applied at each scatterer (metasurface configuration) and can be reconfigured at a modulation rate dictated by either the modulation bandwidth of the metasurface or by the write-speeds achievable by the control architecture, allowing spatial information to be resolved. We focus on electrical tuning mechanisms, because of their potential for subwavelength, independent addressing of scatterers [25]. In Figure 2(a), the detector is schematically illustrated as either in the transmissive or reflective far field of the metasurface. In a practical device, however, the detector should be designed to couple to the near field of the metasurface whenever possible, as an active metasurface coupled to a single-pixel detector in the near field has ‘zero’ added thickness. The system thickness is then defined by the cumulative thickness of the active metasurface and the detection layer, which could enable compact, low-profile single-pixel detector systems with extremely low size, weight and power consumption (SWaP). Whereas in lens-coupled imagers the ratio of lens diameter to focal length limits the FOV, the FOV of an active metasurface imager is limited only by scatterer pitch and the angular sensitivity of scatterer amplitude and phase responses.

Figure 2(b) illustrates a first possible near field coupling mechanism, where light from a certain angle is selectively coupled into a photoactive material. Light intensity can subsequently be extracted from a current measurement. This possibility has already been demonstrated in Kogos et al.’s work [23]. This mechanism, however, imposes constraints on the type of detectors which can be used and may in particular be problematic at longer wavelengths. Alternatively, Figure 2(c) proposes using active modulation to set the angle from which light couples into a waveguide (that is, to set the momentum-matching condition), which itself is coupled to an external detector element.

The choice of coupling mechanism will also affect the design of the active metasurface. While reflective active metasurfaces are more developed at this time, active transmissive architectures [47] are preferable for minimizing device footprints as we anticipate that for many detector technologies, near field coupling between the active metasurface and detector would be simpler in transmission [23]. We expect that further developments in transmissive active metasurface architectures and in on-chip light detection will give rise to more versatile active imaging mechanisms in the future [44], [47].

In the sections that follow, we explore the trade-offs and limitations of an active metasurface imager by modeling the imaging capabilities of a single detector coupled to a local, two-dimensional active metasurface composed of scatterers placed in a rectangular grid. We assume that each scatterer *n* has an addressing-voltage dependent amplitude and phase response, ${\tilde{r}}_{n}=a\left(v_{n}\right){\text{e}}^{\text{i}\psi \left(v_{n}\right)}$ where $a\left(\cdot \right),\psi \left(\cdot \right)$ are the voltage dependent amplitude and phase responses, respectively, and *v*_*n*_ is the control voltage applied to scatterer *n*. We consider the near-monochromatic imaging of a far-field scene and assume no significant interference between different points of a scene over the integration time of our detector. For simplicity, we assume a single polarization and that our detector receives light from a narrow acceptance angle range around a nominal angle *θ* = 0° (Figure 2(a)). Details of our modeling are provided in Supplementary Information 1 and 2.

## 4 Scalability, control architectures, and reconfiguration rates for large-scale apertures

While fabrication of large (mm)-scale active metasurfaces in two dimensions has historically been a major challenge [25], [48], recent fabrication with deep ultraviolet lithography [14] and nanoimprint lithography [49], [50], [51] have demonstrated huge improvements, enabling centimeter-scale, high throughput fabrication of passive metasurfaces. However, scalable fabrication of active metasurfaces faces a significant additional challenge in the form of 2D addressing of subwavelength scatterers.

### 4.1 Scalability of control architectures

**Figure 3:**
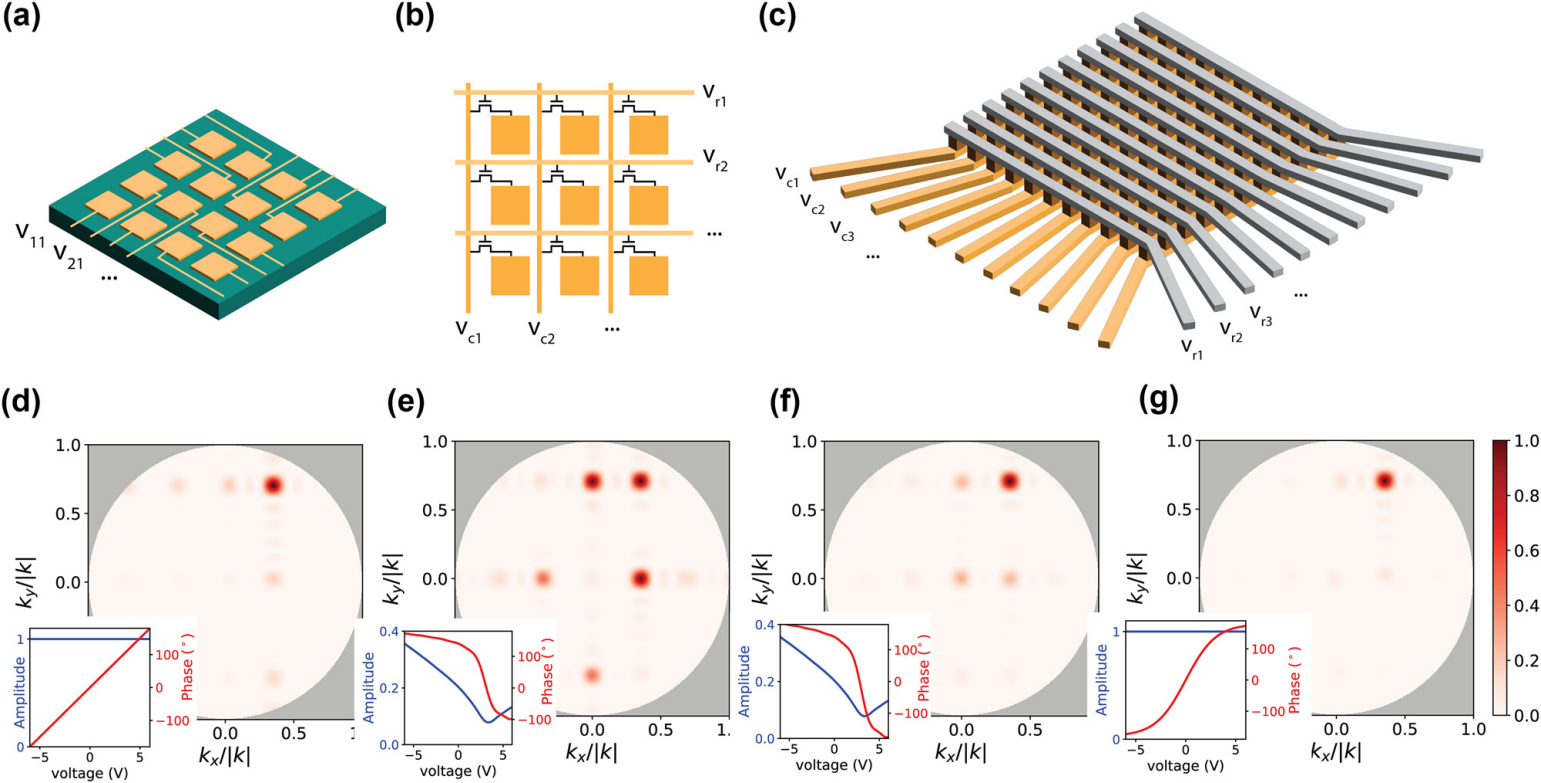
Active addressing of metasurfaces: (a) direct addressing of an *M* row by *N* column rectangular array of scatterers, with *M* × *N* simultaneous signals. (b) Row-column addressing of scatterers for full 2D control, with *M* + *N* simultaneous signals. Switching transistors are used to connect and disconnect scatterers, such that *MN* independent values can be set through bit and wordlines. Scatterer cells maintain their voltage via capacitance (not explicitly illustrated) when disconnected. (c) “Perimeter-control” addressing of scatterers, with *M* + *N* simultaneous signals. Each scatterer $\left(m,n\right)$ is gated by voltage $v_{m}^{\text{row}}-v_{n}^{\text{col}}$ . (d–f) Simulated beam steering to $\left(u_{x},u_{y}\right)=\left(0.4,0.75\right)$ , using the perimeter-control addressing scheme from (c). Simulations assume isotropic radiation, a 32 × 32 scatterers, 13 μm × 13 μm (8.5λ_0_ × 8.5λ_0_) aperture, and the amplitude and phase voltage responses shown in the insets: 360° phase tunability with no amplitude variation (d), experimentally realizable 272° phase tunability with amplitude variation (e), 360° phase tunability with amplitude variation (f), tanh-shaped 360° phase tunability across a narrowed voltage range, with no amplitude variation (g).

We restrict our analysis to electrical addressing, as this approach is the most suitable for realizing active control at the length scales of individual metasurface unit cells, and consider three possible architectures, in decreasing order of complexity and increasing order of scalability. The first architecture is illustrated in Figure 3(a) and is composed of *M* × *N* simultaneous control signals for an *M* row by *N* column rectangular array, each directly connected to a scatterer. While this architecture has been used to demonstrate two dimensional beam steering across small apertures [52], the number of required biasing lines becomes quickly intractable for larger apertures, which are necessary to increase imaging resolution. As such, we would not recommend that this architecture be pursued for imaging applications.

Figure 3(b) presents an alternative approach, row-column tuning, which is commonly used in commercial spatial light modulators consisting of millions of elements and is the same method by which random-access memory (RAM) works in computing. While all *MN* scatterers can be biased with independent voltages following this scheme, these voltages are not all simultaneously applied but rather sequentially set by row (wordline) and column (bitline), using *M* + *N* biasing lines. The voltage value is then held by the unit cell itself either via capacitance, as is the case in dynamic RAM (DRAM), or through logic gates, as is the case in static RAM (SRAM). This row-column tuning approach has been used by Kuznetsov in the experimental realization of a liquid crystal metasurface with 480 × 640 pixels, each 1 × 1 μm^2^ [53]. Moreover, using state-of-the-art fabrication processes, it is possible to have DRAM unit cells as small as 50 × 50 nm^2^ and SRAM as small as 160 × 160 nm^2^ $\left(38\;\text{Mb}/\text{m}{\text{m}}^{2}\right)$ [54], [55]. It is thus conceivable that this technology could scale to much smaller pixel sizes. We refer to a previous perspective for further insights into this addressing method [25].

It should be noted, however, that unlike RAM cells which are designed to hold a single bit, a metasurface with a smooth phase gradient should have a multi-level voltage applied to it. In that case, additional space allowances should be made for a digital to analog converter (DAC) or other required circuits such as latches and drivers. The power cost of setting scatterer voltages and the necessity for thermal dissipation would also further complicate the design. A few factors suggest that this may be possible, particularly with larger metasurface pitches (at longer wavelengths). First, we estimate that 3 bits (8 levels) of information is sufficient for full 2D beam steering applications (Supplementary Information 3), thus allowing for very small DAC form factors. Second, the small size of scatterer elements suggests a low capacitance, which means both a small RC constant and small energies $\frac{1}{2}CV^{2}$ . The RC constant would also be minimized by the proximity of the circuits to the metasurface elements, which limits parasitic resistance. Finally, multilayer electronics might allow us to fit additional electronics in the backplane [56]. However, the viability of this approach depends strongly on metasurface pitch and scatterer design, and the engineering challenge and cost of the system would be significant even in the best case. Additionally, the presence of electronic controls in the backplane would make the implementation of transmissive architectures and on-chip detection prohibitively difficult. Thus, we study and recommend the usage of a third, simpler addressing architecture.

Figure 3(c) illustrates this third, perimeter-controlled architecture, which was first introduced by Davoyan and Atwater for the active tuning of optical phased arrays and metasurfaces [57]. To date, the authors are only aware of two theoretical studies which followed Davoyan and Atwater’s work [58], [59], neither of which focused on system-wide implications of the architecture, or on the effect of scatterer characteristics on achievable control. This architecture directly addresses each row and column with *M* + *N* independent voltages, resulting in a voltage difference $v_{m}^{\text{row}}-v_{n}^{\text{col}}$ applied to the unit cell at row *m* and column *n*. This greatly simplifies the device fabrication, eliminating the need for memory circuits at each unit cell and facilitating the design of transmissive architectures. It also, in principle, allows much faster reconfiguration of the full aperture.

Throughout this paper, we consider an example active metasurface operating at a wavelength *λ_0_* = 1510 nm with a pitch Δ_*x*_ = Δ_*y*_ = 400 nm. This choice is motivated by availability of data for an experimentally demonstrated metasurface at this wavelength. For this metasurface geometry, we simulate the selective coupling (or equivalently, beam steering) of light from wave vector $\vec{k}/\left|k\right|=\left(u_{x},u_{y}\right)=\left(0.4,0.75\right)$ in Figure 3(d)-(g). In each case, the maximum voltage difference $v_{m}^{\text{row}}-v_{n}^{\text{col}}$ across any metasurface element is constrained to be between ±6*V*. Figure 3(d) assumes a so-called ‘ideal’ metasurface response with 2*π* of phase response which is linear in the applied voltage, and no amplitude variation (see inset). Whereas full 2D control over a metasurface with such characteristics would allow total suppression of undesired diffraction orders, this is not the case if constraints on maximum voltage differences are set across all row-column combinations. The constraints apply an effective limit on the accessible phase across elements, as two adjacent rows cannot both achieve a limiting voltage of 6 V (phase of 180°) if they have different voltages applied to them. Nonetheless, the undesired orders remain relatively small.

Figure 3(e) assumes a realizable amplitude and phase response to voltage, with 272° of phase tunability and significant covariation of amplitude and phase, modelled after an experimentally demonstrated ‘realizable’ plasmonic active metasurface on a transparent conducting oxide (TCO) platform (see also Supplementary Information 4) [28]. This realizable metasurface was experimentally demonstrated for a one-dimensional array in reflection; here, we extrapolate the device characteristics to a two-dimensional device. We optimize the row and column voltages to achieve the steering performance in Figure 3(e), which shows significantly increased unwanted diffraction. This leads to later discussed aberrations in recovered images. Nonetheless, even with a highly non-ideal platform, this scheme of addressing is indeed sufficient for image acquisition.

Figure 3(f) assumes a similar amplitude and phase response to Figure 3(e) but with the phase control extended to 360°, leading to significantly improved steering performance. Thus, we find that a perimeter-control addressed metasurface can achieve beam steering performance equal to that of a fully 2D-controlled metasurface, given an improved range of control over individual scatterers. We confirm this in Figure 3(g), where we narrow the voltage range necessary to achieve ∼360° of phase and demonstrate almost fully suppressed diffraction. While the design of said scatterers remains an outstanding challenge, this result provides a path towards fabricable, large-scale apertures.

### 4.2 Reconfiguration rates across large-scale apertures

The rate at which a full imaging aperture can be reconfigured depends on the rate of refractive index modulation achievable within the scatterers (which has been shown to exceed 10 MHz in the previously discussed TCO tunable metasurface and to reach 5 GHz in electro-optic platforms [30], [60]) as well as on the rate at which voltage values can be written to the metasurface. In practical experiments, we also expect to be limited by RC constants within our experimental setup. In this section, we focus on the write times to derive an upper bound to achievable reconfiguration rates, before discussing additional experimental challenges. We consider two cases: a 511 × 511 array of fully 2D row-column addressed scatterers and an easier to realize 255 × 255 perimeter-control addressed array. We assume an array pitch of 400 nm and operation at a wavelength of 1510 nm when calculating the total number of points imaged.

Assuming the target measurement voltages are stored on SRAM with a row-cycle time of 2.7 ns across 72 parallel data line [61] and 3 bits (8 levels) per scatterer voltage, a 511 × 511 array could be fully reconfigured at a rate of 34 kHz (see Supplementary Information 5). This is comparable to the modulation rate achievable by a DMD [31]; unlike a DMD, however, this limit is not fundamental and could be bypassed through a specialized hardware architecture with more parallel data lines. By the same calculations, and assuming 2 bits per row/column voltages which Supplementary Information 3 demonstrates is sufficient for imaging, the 255 × 255 perimeter-control addressed array could be reconfigured at a rate of 26 MHz, enabling very high-speed acquisition of measurements. Storing optimal choices of voltage for each measurement also requires less memory with perimeter-control addressing than with 2D row-column addressing (Supplementary Information 5). With proper design of latches, the digital write times of the circuit are decoupled from the analog time constants (settling time of DACs, drivers and scatterer response), which can also in principle reach MHz rates [62], [63]. Thus, the metasurface reconfiguration time would be limited by the slowest of the full aperture write times, DACs, electronic drivers, and scatterer optical modulation rate.

It should be noted, however, that we expect resistances and capacitances of our control circuit to further limit achievable modulation rates in experimental research demonstrations, even for a perimeter-control addressed device. The design of electrodes, choice of digital to analog converter, and routing of signals may all contribute to an increased RC time constant. In principle, these issues could be resolved through dedicated engineering effort, as they have been in computer architectures.

## 5 Imaging metrics, system comparisons, and outlooks in active metasurface design

In this section, we consider the theoretically achievable performance of lensless, single-pixel active metasurface imagers through conventional imaging metrics, offer a comparison to other existing systems, and comment on the need for improvements of active metasurfaces for this application.

### 5.1 Information limit as a function of aperture size

The simplest method of imaging a scene using an active metasurface is to retrieve the scene point-by-point. The role of the active metasurface in this system is then to direct light from time-dependent target solid angles toward the normal. This task is notably analogous and reciprocal to the well-studied problem of beam steering [64], where incident light is directed to another angle. Conceptually, this approach corresponds to imaging of the scene with a basis of delta functions each aimed at collecting light from a target point in k-space. In practice, our metasurface cannot generate an ideal delta function of coupling, leading to a finite achievable angular (k-space) resolution. We can analytically demonstrate that for a local metasurface with ideal phase and amplitude response, this resolution is diffraction-limited as long as the single-pixel detector can be coupled to a sufficiently small range of angles (Supplementary Information 6).

Active metasurfaces can additionally, in principle, achieve very wide FOV imaging. FOV is generally limited by either the pitch, Δ*x* or Δ*y*, of the subwavelength elements relative to the wavelength of operation, *λ*_0_, or by the efficiency at which scatterers couple to plane waves from large angles. Assuming an approximately isotropic scatterer response, a full 180° FOV can be imaged without aliasing if $\frac{\lambda_{0}}{\Delta x},\frac{\lambda_{0}}{\Delta y}\geq 2$ is satisfied [65]. Achievable FOVs for different pitches are reported in Supplementary Information 7. To demonstrate the feasibility of these wide FOVs, we also include full-wave simulations of a TCO metasurface effectively scattering light incident from up to 71° towards the normal in Supplementary Information 8. We see that, within numerical accuracy, the efficiency of these scatterings is equal to the scattering of normally incident light to large angles.

**Figure 4:**
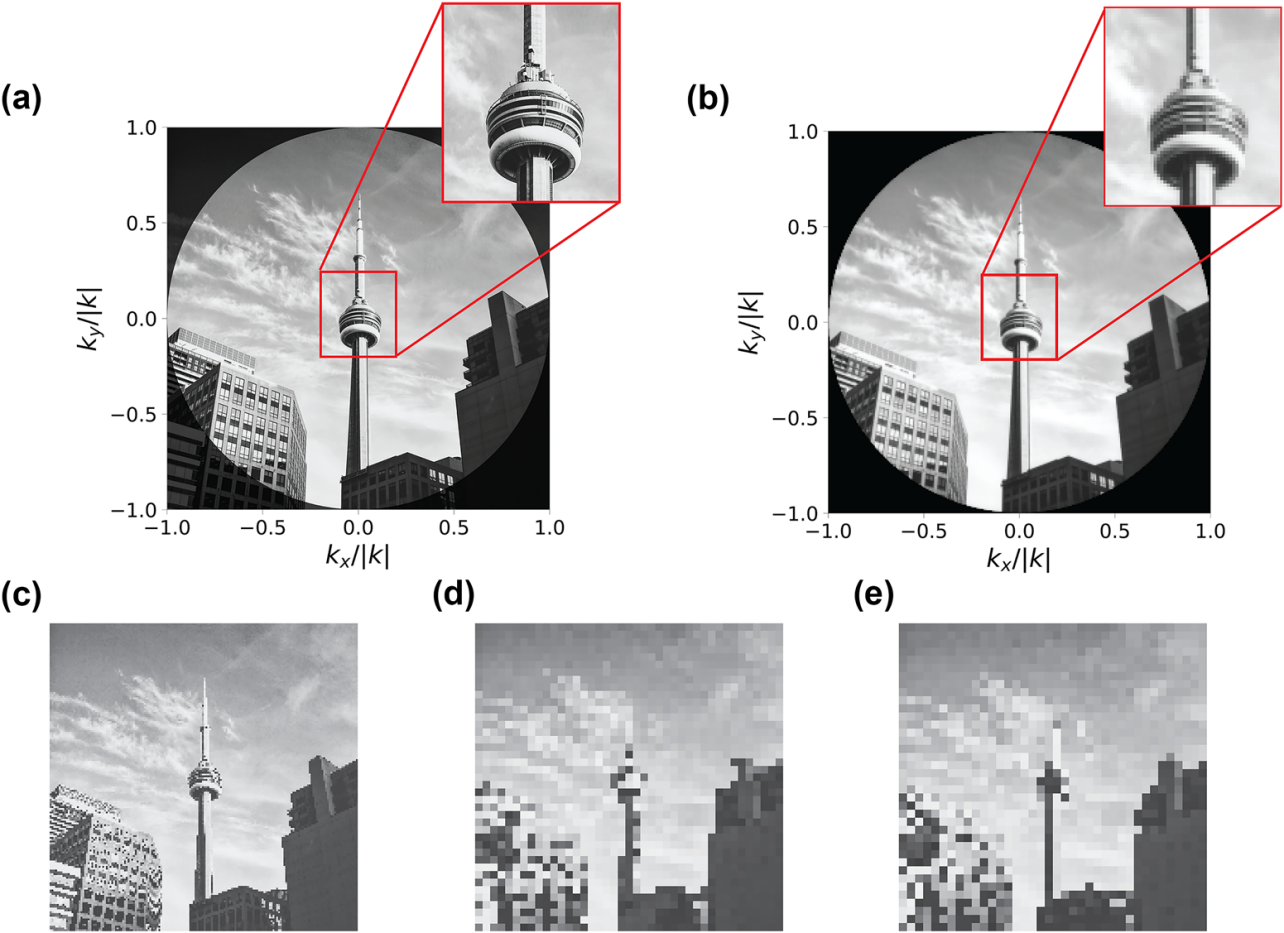
Information acquisition limit comparison: (a) quasi-monochromatic (approximated as a single wavelength) ground truth scene of the CN tower, defined across a full 180° field of view. Picture by Patrick Tomasso on Unsplash. (b) Simulated recovered image from an ideal active metasurface with a 511 × 511 scatterers, 0.2 mm × 0.2 mm (135*λ_0_* × 135*λ_0_*) aperture. 57,609 separate angular measurements are resolved in series, with diffraction-limited resolution. (c–e) Visual comparison of the information limit for a 0.2 mm × 0.2 mm device size which matches (b), where the information is limited by: a CMOS detector array with a 1.2 μm pitch (c), an IR sensor with a 5 μm pitch [66] (d), and a digital micromirror device with a 5.4 μm pitch [67] (e). Images are rescaled computationally with OpenCV in Python and are only meant to be illustrative.

Consider the ground truth image in Figure 4(a). Assuming ideal phase properties and a modest 0.2 × 0.2 mm^2^ (135*λ_0_* × 135*λ_0_*) aperture for our previously described metasurface wavelength and geometry, we find that the high resolution and 180° FOV allows us to resolve ∼60,000 image points with this proposed active metasurface single-pixel imager (Figure 4(b)). In contrast, a CMOS sensor array with a small pixel pitch of 1.2 μm would have only ∼29,000 distinct pixels (Figure 4(c)), a short-wavelength infrared sensor array with a pixel pitch of 5 μm [66] would have ∼1,700 pixels (Figure 4(d)), and a DMD of the same aperture size with a pitch of 5.4 μm [67] would have only ∼1,400 pixels (Figure 4(e)). Thus, we see that for a set device aperture size, owing to its dense pixel array, a hypothetical wide-FOV active metasurface imaging device could resolve more image points than any system which statically couples light into sensor arrays – including the previously discussed metasurface-enabled imaging systems.

In particular, the active nature of the proposed device allows the full aperture size to be used for angularly selective light collection, whereas a passive imager such as Kogos et al.’s compound-eye imager must split its aperture into angle-sensitive pixels. Thus, for a set aperture size, each angle-sensitive pixel is much smaller in a passive device, resulting in a lower diffraction-limited resolution. To successfully exploit the information content advantage of an active device, however, we anticipate that significant improvements may be required in the design of deeply subwavelength actively addressable scatterers, particularly at shorter wavelengths.

### 5.2 Aberrations introduced by limitations of active modulation

Next, we consider aberrations introduced in images by three non-idealities of the active metasurface platforms: covariation of amplitude and phase and limited phase response, directional (dipole) coupling of scatterers to the far field, and constraints of the perimeter-control addressing scheme. We choose to model our array as an array of uncoupled dipoles to study how poor coupling of light at oblique incidences deteriorates imaging performance. Here, we assume that the null of our scatterer dipoles is aligned along the *y* axis. Our methods to select the addressing voltages are described in Supplementary Information 9.

**Figure 5:**
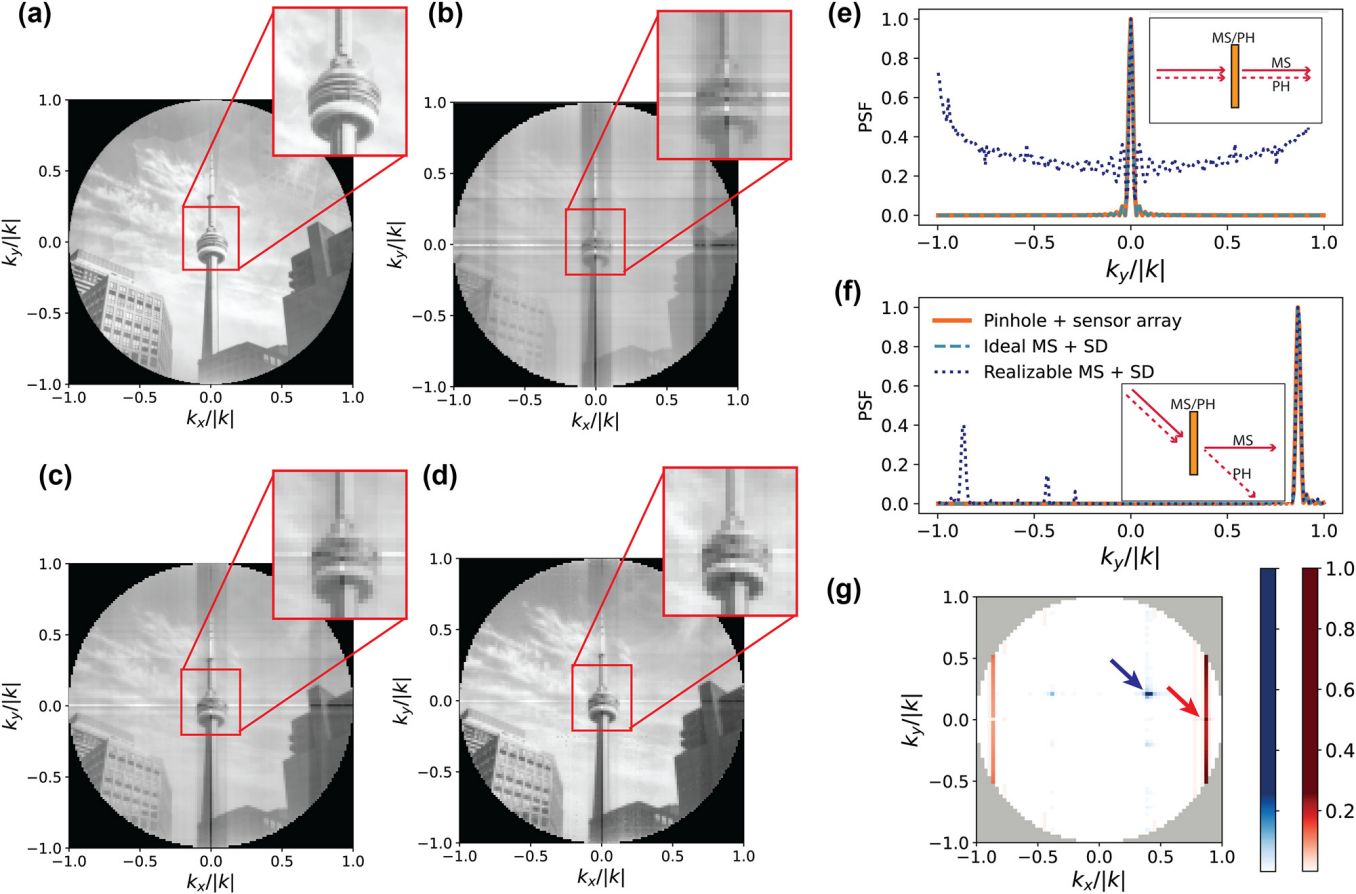
Aberrations in realistic single-pixel, lensless active metasurface imaging: (a) simulated recovered image from a 511 × 511 scatterer realizable metasurface platform with 272° of phase tunability, covarying amplitude and phase, and full 2D control over the addressing voltages (*MN* separately controlled voltages). Pitch and aperture size are assumed to be the same as in Figure 4(b). (b)–(d) Simulated recovered image from a 255 × 255 scatterer realizable metasurface platform controlled by perimeter-control addressed voltages (*M* + *N* separately controlled voltages) with: (b) 272° of phase tunability, covarying amplitude and phase, (c) 360° of phase tunability, covarying amplitude and phase, (d) constant amplitude and 360° of phase tunability across a narrowed voltage range. (e–f) Point spread function (PSF) of the imager described in (a) with a smaller aperture size of 51 μm × 51 μm (dark blue dotted line); of a diffraction-limited pinhole camera (solid orange line); and of an ideal metasurface with isotropic scattering properties, 2*π* phase control, and no amplitude variations (dashed turquoise line). The PSF is plotted along *k*_*x*_ = 0 for a point source originating at $\left(k_{x},k_{y}\right)=\left(0,0\right)$ (e) and $\left(k_{x},k_{y}\right)=\left(0,\frac{\sqrt{3}}{2}\left|k\right|\right)$ (f). The insets schematically depict collection of light by an active metasurface (MS) and pinhole (PH). (g) PSF of the row-column perimeter-control addressed imager in (b) with a smaller aperture size of 51 μm × 51 μm for on-axis (red) and off-axis (blue) points. The arrows indicate the location of the point sources.

Figure 5(a) shows simulated imaging of the ground truth scene (Figure 4(a)), assuming dipole scatterers, a fully 2D-addressable 511 × 511 array of scatterers, and realizable covariation of amplitude and phase [28]. In contrast to the ideally recovered image (Figure 4(b)), these realistic features lead to systematic aberrations in the image (Figure 5(a)). These aberrations include a decrease in the contrast of the image, particularly at large *k*_*y*_ to which our scatterers couple poorly, as well as ‘ghost images’ at reflected locations which can be most clearly seen as the outline of buildings in the sky. They can be understood via the angle-dependent PSFs of the imager. Light from a point source at 0° is coupled into each measurement to varying extent (Figure 5(e), dark blue dotted line), lowering the contrast achievable in Figure 5(a) – this coupling dominates over the desired coupling of oblique incidence light at large *k*_*y*_, as signal couples less efficiently to the dipole scatterers along this axis. Additionally, point sources at oblique angles couple into both the intended $\left(k_{x},k_{y}\right)$ measurement and the $\left(-k_{x},-k_{y}\right)$ measurement (Figure 5(f)) – this explains the ‘ghost images’ in Figure 5(a). In contrast, a fully 2D addressable active metasurface with constant amplitude response and 2*π* of phase control (dashed turquoise line) has the same PSF as an ideal pinhole (solid orange line) and yields the imaging performance shown in Figure 4(b).

We also consider an easier to manufacture device in Figure 5(b)–(d), with a smaller 255 × 255 array of perimeter-control addressed scatterers, each with dipole coupling. First, we note a lower resolution in comparison to Figure 5(a); this is only a consequence of the reduced aperture size, not of the addressing method. Figure 5(b) is simulated with 272° of phase control, whereas Figure 5(c) assumes 360° of phase control and both assume covarying amplitude and phase. Figure 5(d) assumes a more ideal scatterer response, with constant amplitude and nearly 360° of antisymmetric phase response, as in Figure 3(g). Each figure exhibits horizontal and vertical stripe aberrations, which is consistent with the simulated 2D PSF associated with Figure 5(b) (Figure 5(g)), which shows that light from on-axis points is detected even by off-axis points (red PSF). However, as suggested by Figure 3, these aberrations disappear as scatterer response improves.

Unlike lens-based systems, which typically have ‘broadening’ aberrations in which the collected image becomes blurry, these aberrations introduce predictable effects with diffraction-limited width. Thus, we anticipate that they will be straightforward to correct with computational post-processing (see also Supplementary Information 10). Note that this type of aberrations is also found in passive angular-coupling imagers [23], and is characteristic of a ‘beam steering’ approach to light collection. We expect that improvements in scatterer design, specifically increased phase control and reduced amplitude variation, will also greatly improve performance.

### 5.3 Signal-to-noise ratio (SNR) implications of angularly selective coupling

Whereas all the light incident from the FOV can generally be assumed to arrive at the detector array in a lens-coupled system, this is by necessity not the case in single-pixel imaging systems, which acquire spatial information precisely through selective rejection of light from certain object points. While some single-pixel imaging setups such as the lens-coupled DMD system in Figure 1(c) can in principle harvest up to half of the incident light from the FOV for each measurement, this is not the case for imagers which operate by angular selection of light, whether active or passive.

For a fixed acquisition time *τ*, aperture area *A*, and resolution, passive and active angularly selective imagers have the same fundamental limit in light-usage efficiency. Consider a passive aperture, which is divided into *N* ^2^ pixels of area ∼*A*/*N* ^2^ which each retrieve light from a separate solid angle for a time interval *τ*, and an active aperture of area *A* that acquires each measurement only for time *τ*/*N* ^2^. The total incident power on both apertures is the same – if we assume same-resolution imaging (according to which a same proportion of incident light is used for detection), the photon shot noise limited performance should be the same. The shorter integration time for the active aperture may also reduce other noise sources such as dark noise. However, if we choose to use the improved resolution achievable by an active aperture, the SNR will decrease. This is further discussed in Section 5.4. Thus, the most significant penalty a lensless, single-pixel active metasurface imager suffers for its combined small form factor and high information limit is a deterioration in SNR for an acquired image.

**Figure 6:**
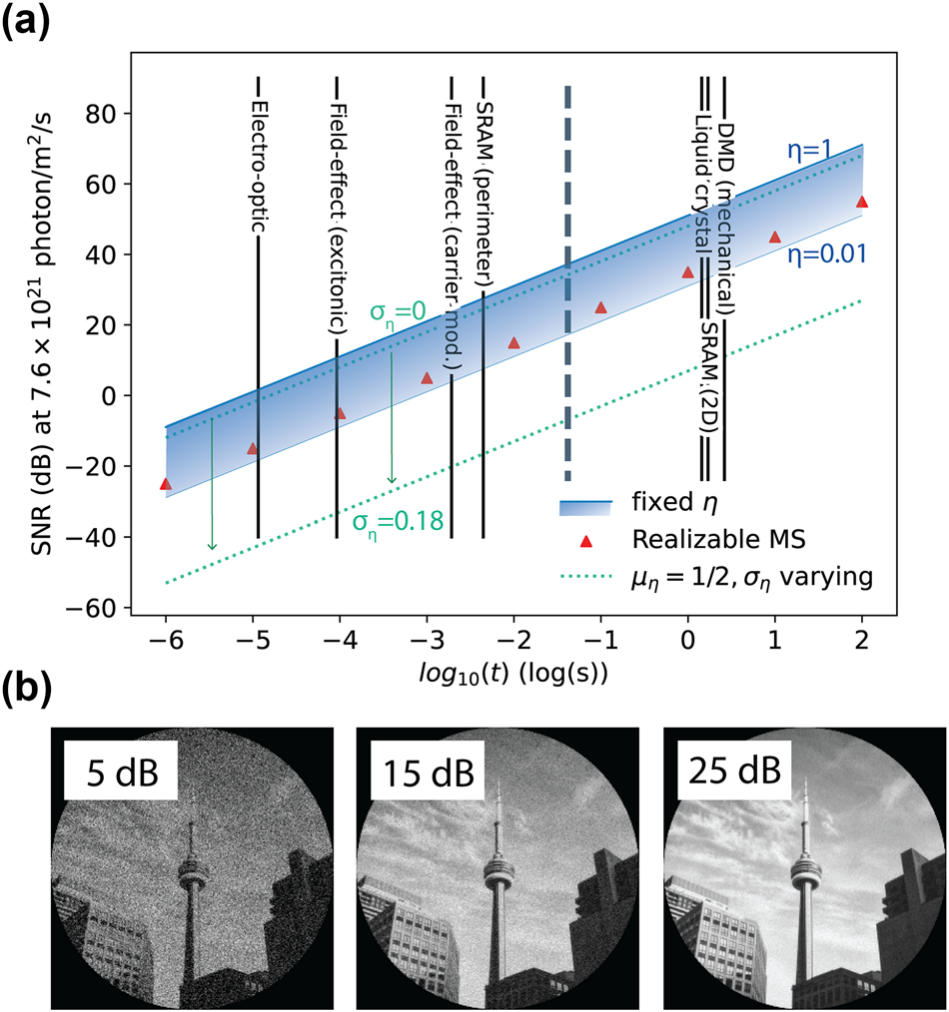
Effects of loss, SNR, and modulation rates on image acquisition time: results assume a quasi-monochromatic light source with a photon flux of $7.6\times 10^{21}\frac{\text{photons}}{{\text{m}}^{2}\;\text{s}}$ , an aperture size of 0.2 mm × 0.2 mm, a single detector with a uniform, diffraction-limited square acceptance in k-space, and 57,609 measurements. (a) SNR (dB) as a function of the logarithm of time *t*, for various measurement loss conditions. The blue shaded region assumes a fixed efficiency across all measurements, from 1 to 0.01. The dotted green lines demonstrate the effect of variance in measurement efficiency, where the SNR at large variance is dominated by the high-loss measurements. The red triangles indicate SNR for the realizable metasurface [28]. The black vertical lines indicate the modulation-speed limited imaging time for different modulation mechanisms and control architectures. The vertical dashed blue line shows the acquisition time below which video-rate imaging is achievable. (b) Examples of the retrieved image at various shot-noise limited SNRs. The Poisson shot noise is approximated as normal noise.

This has a number of implications. First, tasks which do not require operation in low-light conditions – for example, industrial inspection [9] – appear as promising applications. Alternatively, we can consider applications which aim not to recover a full image but rather to isolate signal from a small (potentially time-varying) position in space, in which case the angular selectivity becomes beneficial to reducing background noise. From a design standpoint, this disadvantage is offset by the possibility of integrating more sensitive single detectors, which might not exist as sensor arrays. This drawback also motivates the development of low-loss (non-plasmonic) active metasurfaces [37], [47], since in most experimentally realized active metasurfaces to date, it has been difficult to achieve both high modulation rates and low losses in the same device. We consider the trade-off between modulation rates and device losses in Figure 6(a), assuming a shot noise limited system to focus only on the impact of the active metasurface element. Details of the calculation are provided in Supplementary Information 11. Note that the SNR presented in Figure 6 is an ‘image-wide SNR’ defined as the sum of squared signal power over the sum of squared noise power [68] – this is different from a per-measurement SNR (signal power over noise power).

Figure 6(a) assumes an average photon flux of 7.6 × 10^21^ photons/m^2^/s (equivalent to 1 sun or 1,000 W/m^2^ of power at a wavelength of 1510 nm), a 0.2 × 0.2 mm^2^ aperture, a diffraction-limited detection window, and 57,609 measurements collected point-by-point from diffraction-limited measurements. We consider two possible mechanisms for losses: material absorption, and poor coupling of light into scatterers which results in some light not being steered. We first consider how losses which are uniform across all measurements affect the SNR, through the blue shaded region which indicates SNR as a function of time for systems with an efficiency between 100 % and 1 %. As expected, we see 10 dB of loss for each order of magnitude drop in metasurface intensity scattering efficiency. Next, we consider the case where loss varies across measurements through the dotted green lines. Losses for each measurement are sampled from a normal distribution with mean efficiency 0.5 and a standard deviation of 0 (top line) and 0.18 (bottom line), then clipped to fall within a physical range $\eta \in \left(\varepsilon ,1-\varepsilon \right),\varepsilon \ll 1$ . We find that in systems where loss varies with the measurements, the SNR is dominated by the measurements with the highest loss. Finally, we consider the previously discussed ‘realizable’ TCO plasmonic active metasurface [28], assuming dipole coupling of scatterers to the far field and find an expected SNR (shown as red triangles in Figure 6(a)) of ∼16 dB below that of the ideally achievable SNR.

Given an expected photon flux, target aperture size, SNR, and image resolution, these calculations can help select which state-of-the-art active metasurface platform is most suited to a desired application. Figure 6(a) shows the material modulation-rate-limited and addressing architecture-limited image collection times for different active metasurface platforms and addressing architectures as solid vertical lines. The values for currently achievable modulation rates are taken from Ref. [25] for metasurface platforms, Ref. [31] for DMDs, and are derived in Supplementary Information 5 for the addressing architecture write-times. We find that under the illumination conditions plotted, a perimeter-control addressed realizable TCO metasurface can collect an image with an SNR of less than 35 dB faster than a liquid crystal metasurface due to its faster modulation rate. However, when aiming to retrieve high SNR images (*e.g.,* SNR > 40 dB), or assuming a lower irradiance, a liquid crystal metasurface becomes preferable due to its lower losses.

These image acquisition rates also limit the system’s ability to image dynamically. To acquire video-rate imaging of 24 frames per second at the resolution assumed in Figure 6, each image would have to be acquired in ∼42 ms, a threshold shown in Figure 6(a) as a dashed vertical blue line. At the chosen illumination, this corresponds to an SNR of 37 dB for an ideal active metasurface and of 21 dB for our realizable plasmonic metasurface – for this image resolution, the required modulation rates preclude the use of liquid crystal or DMD modulation platforms. Images with 5–25 dB of noise (modelled as normal noise) can be viewed in Figure 6(b), for reference.

### 5.4 Fundamental trade-offs in SNR, resolution, and acquisition time

The stated bounds on the number of resolvable points and the resolution so far assume a diffraction-limited imaging system where the imaging characteristics are dominated by the optical response of the metasurface. However, the effective k-space detector width (angular acceptance range) of the coupled single-detector also affects the system PSF, which broadens proportionally to the detector width once the resolution drops below the diffraction limit. Additionally, for a fixed aperture size, the rate of photon arrival at the detector increases proportionally to the detector k-space area and the number of measurements to recover the full FOV decreases with resolution. Then, increasing the detector angular acceptance range can improve the SNR achievable by each measurement across the full image acquisition time.

Thus, the k-space detector area mediates a fundamental trade-off between the number of resolvable points and the per-measurement SNR in a lensless, single-pixel active metasurface imager. Here, we discuss not the image-wide SNR in Section 5.3 but rather the simpler metric of signal power over noise power. Trade-offs in the image-wide SNR are presented in Supplementary Information 12. With this definition, for a fixed total image acquisition time where the SNR is dominated by shot noise ( $SNR\propto \sqrt{N}$ , where *N* is the number of collected photons), we find that $SNR\propto \frac{1}{N_{p}}$ , where *N*_*p*_ is the total number of points imaged (Supplementary Information 12). In contrast, the SNR of a conventional lens-coupled sensor array follows $SNR\propto \frac{1}{\sqrt{N_{p}}}$ (Supplementary Information 12), which is a more favourable scaling. It is therefore worth noting that light collection efficiency and SNR are likely to eventually limit the resolutions achievable by the lensless active metasurface single-pixel imager that we describe in this text.

## 6 Discussion

### 6.1 Other active metasurface-enabled imaging modalities

**Figure 7:**
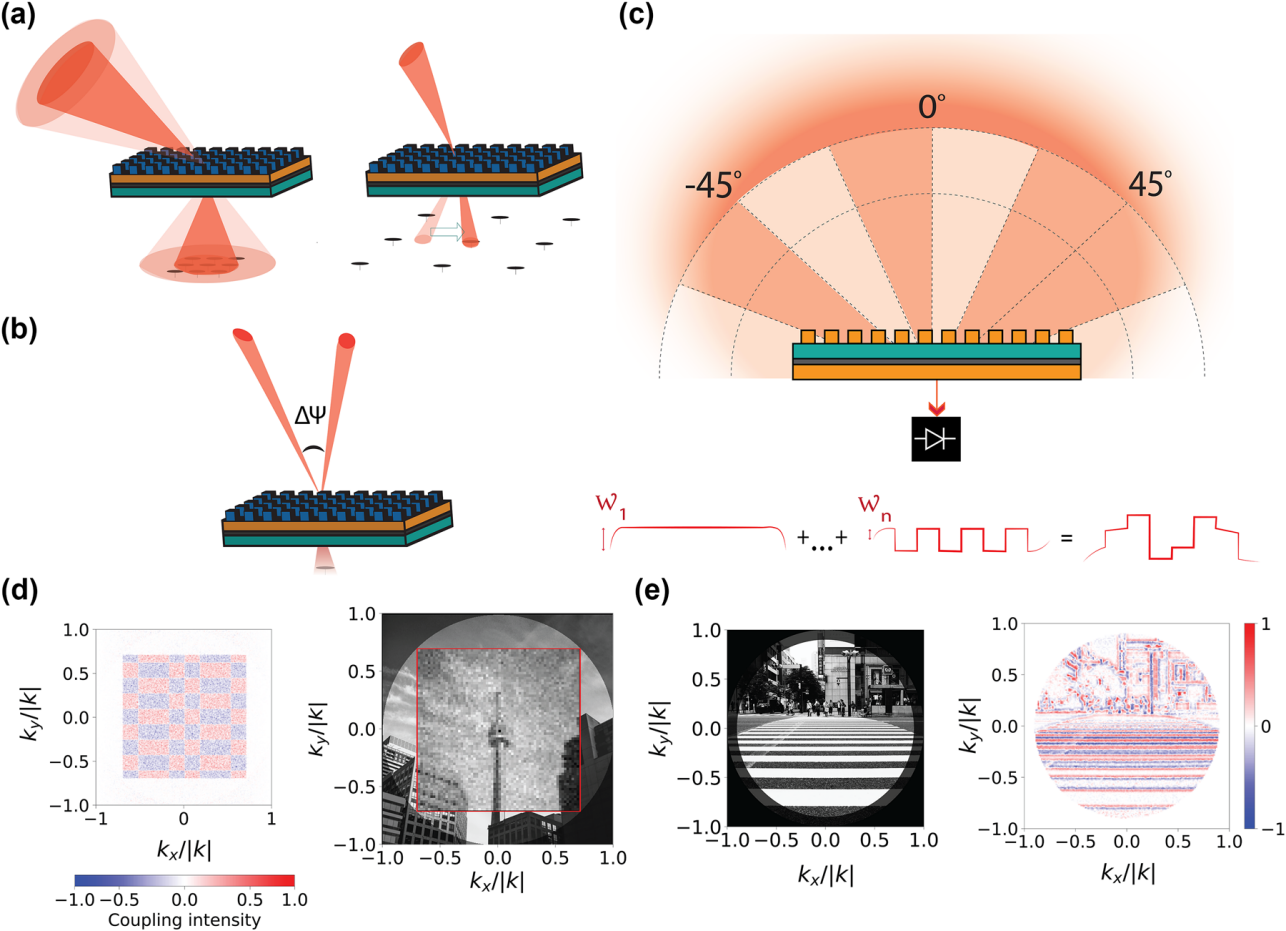
Other imaging modalities for active metasurfaces: (a) multi-pixel imaging. An active metasurface could increase the field of view of a system with a small detector array (left) or the resolution of a system with a sparse detector array (right), through sequential measurements in time. (b) Phase-sensitive imaging. The phase-conserving capabilities of an active metasurface can be used to interfere light collected from different angles of a time-coherent scene. (c) General schematic representation of angularly selective light collection with an active metasurface. Images can be reconstructed from arbitrary choice of basis, defined in the incident k-space (schematically shown in darker orange). Measurements *w*_*n*_ give us the weight of basis element *n* in the image reconstruction. (d) Map of coupling intensity between the incident light and the detector for an example Hadamard basis element (left) used to create the reconstructed image (right). The weight of each basis element is found through the difference of two measurements, one corresponding to the positive coupling and the other to the negative coupling. Image recovery is simulated assuming 345 × 345 scatterers, corresponding to a 0.14 mm × 0.14 mm (91*λ_0_* × 91*λ_0_*) aperture size, imaged with a 4,096-element Hadamard basis. Realizable amplitude and phase characteristics are assumed. (e) Simulated single-pixel edge-detection of a quasi-monochromatic ground truth scene (left, picture by Daryan Shamkhali on Unsplash), with an active metasurface imaging device. The bright circle covers a 128° FOV and is the part of the scene over which edge detection is performed. The dimmer circle extends to a 180° FOV. The edge-detection (right) is performed by selecting voltages to make the system PSF into a Laplacian of Gaussian kernel, through the difference of a positive and negative measurement.

Our discussion has so far focused on the coupling of an active metasurface to a single detector. However, the principle of active metasurface modulation can be applied to a broad range of architectures and be used to control different degrees of freedom of light. Coupling an active metasurface to a small or sparse detector array could enable an information density exceeding the Nyquist limit of the detector array itself. The FOV of a small detector array could be increased without changing the distance to the metasurface aperture by reconfiguring the metasurface to sequentially couple different areas of solid angle onto the detector array (Figure 7(a), left). The resolution of a sparse detector array could similarly be improved by rastering of diffraction-limited light across each pixel (Figure 7(a), right). This would lead to a higher SNR for a set acquisition time than we previously described, at the expense, however, of device compactness.

Moreover, active imagers could be designed to exhibit the same multifunctional control over degrees of freedom as metalenses. For example, a metasurface could be designed to couple only one polarization of light into a detector element. Alternatively, while this work has assumed no phase coherence in time between different points of a scene, active metasurfaces can conserve phase properties of light. Then, it may be possible to perform phase-imaging by selectively interfering light from different angles (Figure 7(b)). Further studies should be performed to determine the degree of control over metasurface scatterers which would be required for this application. Extending this work to multiple wavelengths would also allow a richer range of applications.

In time-critical applications, it may be useful to speed up acquisition time by extracting key information from the scene in significantly fewer measurements by coupling in far field light from multiple angles at a time, as conceptually illustrated in Figure 7(c) through the darker shaded regions. In Figure 7(d), we simulate image recovery in only 8,192 measurements using a k-space Hadamard basis. An example basis element is provided on the left, and the reconstructed image is on the right. In Figure 7(e), we demonstrate single-pixel edge detection by configuring the far field PSF of our function into a second derivative kernel, retrieving an edge-image (right) from a ground truth image (left). We discuss these results further in Supplementary Information 13 and describe the image recovery algorithms in Supplementary Information 14. The ability of active metasurfaces to generate arbitrary coupling between the far field and the detector additionally indicates that this imaging system may be compatible with compressed sensing, enabling image recovery in significantly fewer measurements [11]. However, these more complex measurements require a fully 2D configurable metasurface aperture and are not generally compatible with a perimeter-control addressing architecture; as such, large-aperture demonstration of these concepts will require additional development in addressing technology.

### 6.2 Choice of active metasurface platform

The above analysis directly ties features of metasurfaces to imaging metrics. In general, the following features are desirable to high-speed image acquisition, good resolution, high SNRs, and minimizing aberrations: high efficiency, isotropic antenna factor (coupling) across the FOV of interest, high modulation frequencies, 2*π* phase control, and large aperture sizes. We refer the reader to other reviews which cover the state-of-the-art on these objectives [25], [69], [70]. This raises the question of which active modulation mechanisms and types of scatterer materials are most promising in imaging.

Plasmonic platforms offer a number of advantages – plasmonic scatterers can be designed as relatively omnidirectional such that light collection is enabled across a wide range of coupling angles and can achieve deeply subwavelength pitches with minimal inter-scatterer coupling thanks to their small mode volumes (Supplementary Information 8) [28], [64], [71]. This allows effective suppression of unwanted orders over a wide FOV. However, they are also unavoidably lossy; the reflective plasmonic metasurface which we use as a realizable example in our discussion has an absorption loss between 87 % and 99 % depending on the applied voltage (Supplementary Information 4). Such losses dramatically limit the applications of an active metasurface imager.

Low-loss dielectric platforms are increasingly emerging as promising alternatives to plasmonic metasurfaces, compatible with low-loss, transmissive operation. Liquid crystal active metasurfaces are the most mature of such platforms, having demonstrated multispectral continuous 2*π* phase control over individual pixels, with absolute efficiencies above 40 % [72], and beam steering capability across a 22° FOV [73]. Unfortunately, they also suffer from relatively low modulation rates due to the physical switching time of molecules ( ≤40kHz ) and from significant crosstalk as the metasurface pitch is reduced below 1 μm [25], limiting the possible rates and FOV of operation.

A number of alternate approaches have shown promise in overcoming these limitations. Iyer et al. demonstrated optical-pump based ultrafast dynamic steering of 1.25 μm incoherent emission up to 70° with a GaAs resonator metasurface with a pitch of 400 nm [74]. Forouzmand and Mosallaei proposed designs for carrier-injection modulation of silicon nanobars with a theoretical transmission of above 60 % for a pitch of 575 nm and 215° of phase control [75]. Sokhoyan et al. have reported designs for high-Q devices which full wave simulations suggest are capable of a large degree of phase tuning with modest thermo-optic shifts in refractive index, capable of deflecting light to ±30° [47]. Finally, increased interest in electro-optic materials may soon lead to the realization of new electrically-addressable low-loss platforms, capable of wavefront shaping at MHz or GHz modulation rates [60], [76].

A number of challenges remain to be overcome in these platforms. While the lower index changes achievable in dielectric platforms can be overcome with higher-Q modes [47], high-Q designs tend to exhibit increased angular sensitivity when compared to plasmonic modes. This is a challenge in wide FOV applications, which must be addressed with proper design of scattering elements. In their demonstration of high quality factor passive metasurfaces, Hail et al. observe a spectral shift of less than 2 nm across a 10° change in incidence angle [77]. They also demonstrate high-Q metalenses with numerical apertures of up to 0.8, showcasing that the studied scatterer design is capable of coupling to oblique incidences. Nevertheless, achieving a full 180° FOV with active dielectric scatterers will require further development in the field. Additionally, it should be noted that the larger mode volumes of dielectric Mie modes lead to increased inter-element coupling. This does not necessarily prevent efficient steering but does complicate steering calculations [47]. Nonetheless, we are optimistic that as material synthesis methods and device designs mature, a number of these challenges will be addressed by the field.

### 6.3 Areas of interest for applications of the technology

We believe that active metasurface imagers will be most useful in applications requiring high resolution and FOV and one or both of the following: a very small form factor and the use of few detectors.

In particular, the very small form factor of an active metasurface imager could be used in applications such as surveillance, or endoscopy in medical imaging. In medical imaging in particular, a metasurface’s potential to control additional degrees of freedom such as polarization could be useful, allowing for better contrast in tasks such as cancer detection [78].

The ability of an active metasurface imager to be operated in a single-pixel configuration also opens up possibilities in time-of-flight imaging for precise depth mapping [79], in short-wave infrared for imaging through scattering media such as smoke [80], or in terahertz imaging applications where the cost, size, and operating temperatures of THz sources and detectors hinders commercialization in applications such as cancer diagnosis, artwork inspection, and semiconductor wafer inspection [9]. These longer wavelength applications additionally have the benefit of facilitating subwavelength fabrication and are likely where the technology would see the most success.

## 7 Conclusions and perspective

While metasurface-enabled imaging has led to the creation of compact systems capable of controlling various degrees of freedom of light (phase, polarization, wavelength), there remains a gap in the literature when it comes to very low form-factor, low-pixel count imagers. Simultaneously, current and anticipated advances in the design of active metasurfaces – including the use of low-loss dielectric materials [47], development of transmissive architectures [47], demonstrations of gigahertz rate modulation [60], and proposals for scalable perimeter-control [57] – are making complex, high-speed control of incident light increasingly achievable. We hope that these advances will be used to enable new, active methods of imaging.

We have theoretically demonstrated that a single detector pixel coupled to an active metasurface can acquire more independent measurements of a scene than the number of pixels available on a same-sized CMOS sensor – this makes active methods uniquely well-suited to applications requiring compact, high-resolution and wide FOV imaging. Our proposed imaging approach, however, suffers from a fundamental trade-off between resolution and achievable SNR, at least in the single-pixel configuration; this motivates extending our analysis into other imaging modalities such as multi-pixel imaging, and into the design of low-loss, dielectric metasurfaces. The ability of active metasurfaces to recover high-resolution and wide FOV information, however, depends on our ability to scale deeply subwavelength pixels to large aperture sizes.

To this end, we numerically demonstrate that large-scale imaging apertures can be addressed through a ‘perimeter-control’ architecture which requires only $O\left(N\right)$ control signals for an aperture with *N* ^2^ scatterers, by gating each element by the difference between the row and column voltages. This not only presents a major reduction in fabrication complexity but also allows a factor of *N* speedup in writing the control voltages, while reducing memory requirements for voltage configurations by the same factor. We find that to maintain a fixed steering performance, a system which uses perimeter-control addressing rather than a fully 2D voltage configuration requires improved phase control over individual elements. This remains a significant challenge. However, this challenge is decoupled from the scale of the aperture, unlike memory requirements and the time to configure a full aperture; as such, we expect that perimeter-control addressing will be a key enabler of large (mm to cm) active metasurface apertures.

This work highlights that with proper choice of control architecture, scatterer design, and modulation method, active metasurfaces have the potential to be useful platforms for compact, spatially resolved imaging of degrees of freedom such as polarization and phase. The use of an active device would in particular enable the use of specialized detector elements which are not available in large scale arrays [81]. We thus anticipate that active metasurface imaging will in the future find industrial applications in fields such as LIDAR technologies, medical imaging, and industrial inspection, as well as in specialized scientific applications.

## Supplementary Material

Supplementary Material Details
