# Supplementary material for: Active metasurface designs for lensless and detector-limited imaging
Source: Nanophotonics. 2025 May 5;14(23):3871–88. doi: 10.1515/nanoph-2024-0704 (PMC12617706; doi:10.1515/nanoph-2024-0704)
Supplement: Supplementary file 1 — Supplementary Material Details [file j_nanoph-2024-0704_suppl_001.docx]

**Active metasurface designs for lensless and detector-limited imaging**

Julie Belleville, Prachi Thureja, and Harry A. Atwater*

Thomas J. Watson Laboratories of Applied Physics

California Institute of Technology

Pasadena, CA 91125

[jbellevi@caltech.edu](mailto:jbellevi@caltech.edu), [pthureja@caltech.edu](mailto:pthureja@caltech.edu), [haa@caltech.edu](mailto:haa@caltech.edu)

***Contact information for corresponding author:** [haa@caltech.edu](mailto:haa@caltech.edu), +1 (626) 395-4100

**SUPPLEMENTARY INFORMATION**

**1. Formalism for scattered intensity**

We model the imaging capabilities of a single detector coupled to a local, two-dimensional active metasurface composed of scatterers placed in a rectangular grid. We consider the near-monochromatic imaging of a far-field scene and assume no significant interference between different points of a scene over the integration time of our detector. For simplicity, we assume a single polarization. Under these assumptions, the photon flux per unit k-space arriving at a detector, $I_{detected}(\vec{k}_{out})$, coupled to the outbound wavevector $\vec{k}_{out}=k_{out,x}\hat{x} +k_{out, y}\hat{y}$ (taken to be $\vec{k}_{out}=\vec{0}$ in our simulations) can be written as

$$\begin{aligned} I_{detected}\left( \vec{k}_{out} \right)=\int_{\frac{\vec{k}_{in}}{\left| k_{in} \right|}\leq1} I_{in}\left( \vec{k}_{in} \right)\left| G\left( \vec{k}_{out}, \vec{k}_{in} \right) \right|^{2}\left| A\left( \vec{k}_{in}-\vec{k}_{out} \right) \right|^{2}d\vec{k}_{in}\#\left( S1 \right) \end{aligned}$$

where $I_{in}\left( \vec{k}_{in} \right)$ is the photon flux per unit k-space arriving at the metasurface, , $G\left( \vec{k}_{out},\vec{k}_{in} \right)=g\left( \vec{k}_{out} \right)g(\vec{k}_{in})$describes the complex field coupling between inbound wavevector $\vec{k}_{in}$ and outbound wavevector $\vec{k}_{out}$ (see Fig. 1d) for a single metasurface scatterer, with $g(\vec{k})$ being the antenna factor [1], and $A\left( \vec{k}_{in}-\vec{k}_{out} \right)$is the array factor

$$\begin{aligned} A\left( \vec{k}_{in}-\vec{k}_{out} \right)= \sum_{n} a_{n}e^{i\psi_{n}}e^{i\left( \vec{k}_{in}-\vec{k}_{out} \right)\cdot\vec{r}_{n}}\#\left( S2 \right) \end{aligned}$$

where $\vec{r}_{n}=\left( x \hat{x}+y \hat{y} \right)$ is the position of scatterer $n$, and $a_{n}(v_{n})$ and $\psi_{n}(v_{n})$ are the amplitude and phase response, respectively, subject to voltage $v_{n}$. The array factor is the Fourier transform of the metasurface configuration and encodes the far field interference between uncoupled scatterers. We assume that the phase response and material losses of scatterers can be approximated as angle-independent over the FOV of interest. This assumption is generally valid across a wide FOV for metasurfaces with a lower quality factor, as in the case of an exemplary plasmonic TCO metasurface discussed in the work. In this work, we adopt the convention of indexing our plane waves by their in-plane wavevector components only, because the normal wavevector component $k_{z}$ is fully constrained by our near-monochromatic assumption.

This array factor formalism assumes negligible coupling between adjacent elements. We begin by assuming that the Green’s function response of the metasurface to a plane wave $\vec{k}_{in}$ can be described as $G\left( \vec{k}_{out}, \vec{k}_{in} \right)A\left( \vec{k}_{in}-\vec{k}_{out} \right)$, up to normalization, such that the far field amplitudes scattered from the metasurface are

$$\begin{aligned} E\left( \vec{k}_{out} \right)= \int_{\frac{\vec{k}_{in}}{\left| k_{in} \right|}\leq1} E_{in}\left( \vec{k}_{in} \right)G\left( \vec{k}_{out}, \vec{k}_{in} \right)A\left( \vec{k}_{in}-\vec{k}_{out} \right)d\vec{k}_{in}\#\left( S3 \right) \end{aligned}$$

$$\begin{aligned} H\left( \vec{k}_{out} \right)= \int_{\frac{\vec{k}_{in}}{\left| k_{in} \right|}\leq1} H_{in}\left( \vec{k}_{in} \right)G\left( \vec{k}_{out}, \vec{k}_{in} \right)A\left( \vec{k}_{in}-\vec{k}_{out} \right)d\vec{k}_{in}\#\left( S4 \right) \end{aligned}$$

Considering integration times $\tau$ greater than $T=\frac{2\pi}{\omega}$, where $\omega$ is the frequency of light. We write the time-averaged Poynting vector as

$$\begin{aligned} S\left( \vec{k}_{out} \right)=\frac{1}{2}E_{detected}\left( \vec{k}_{out} \right)\times H_{detected}^{*}\left( \vec{k}_{out} \right)\#\left( S5 \right) \end{aligned}$$

$$\begin{aligned} S\left( \vec{k}_{out} \right)=\frac{1}{2}\left( \int_{\frac{\vec{k}_{1}}{\left| k_{1} \right|}\leq1} E_{in}\left( \vec{k}_{1} \right)G\left( \vec{k}_{out}, \vec{k}_{1} \right)A\left( \vec{k}_{1}-\vec{k}_{out} \right)d\vec{k}_{1} \right) \times\left( \int_{\frac{\vec{k}_{2}}{\left| k_{2} \right|}\leq1} H_{in}\left( \vec{k}_{2} \right)G\left( \vec{k}_{out}, \vec{k}_{2} \right)A\left( \vec{k}_{2}-\vec{k}_{out} \right)d\vec{k}_{2} \right)^{*}\#(S6) \end{aligned}$$

$$\begin{aligned} S\left( \vec{k}_{out} \right)=\frac{1}{2}\int_{\frac{\vec{k}_{1, 2}}{\left| k_{1,2} \right|}\leq1} {[E}_{in}\left( \vec{k}_{1} \right)\times H_{in}^{*}\left( \vec{k}_{2} \right)]G\left( \vec{k}_{out}, \vec{k}_{1} \right)G^{*}\left( \vec{k}_{out}, \vec{k}_{2} \right) A\left( \vec{k}_{1}-\vec{k}_{out} \right)A^{*}\left( \vec{k}_{2}-\vec{k}_{out} \right)d\vec{k}_{1}d\vec{k}_{2}\#\left( S7 \right) \end{aligned}$$

Consider now a time $\tau\gg\frac{2\pi}{\omega}$. This will be true for integration times of interest in imaging. In this case, we can average $S$ over the time $\tau$

$$\begin{aligned} S_{detected}\left( \vec{k}_{out} \right)=\frac{1}{\tau} \int_{t-\tau}^{t} S\left( \vec{k}_{out},t^{'} \right)dt^{'} \#\left( S8 \right) \end{aligned}$$

While the active metasurface modifies a narrowband phase and amplitude response, we assume that the incident light itself is not fully monochromatic, such that it is incoherent in time over the integration time of the detector. This can be justified by the fact that even a narrow bandwidth of operation for an active metasurface remains measured in nanometers [2]—at an operating wavelength of $1510 nm$, a $1nm$ bandwidth is equivalent to $\geq100 GHz$. Thus, there is a significant regime of operation in which light can be treated as monochromatic for our calculations, but in which there is no coherence between light from different points. Then, assuming that each point of our far field is of constant intensity but time incoherent, we note that the cross product of fields from different far field points average to zero on the timescale of $\tau$. That is,

$$\begin{aligned} \frac{1}{\tau} \int_{t-\tau}^{t} {\frac{1}{2}E}_{in}(\vec{k}_{1}, t')\times H_{in}^{*}\left( \vec{k}_{2}, t^{'} \right)dt'=S_{in}\left( \vec{k}_{1} \right)\delta\left( \vec{k}_{1}-\vec{k}_{2} \right)\#\left( S9 \right) \end{aligned}$$

Then, Eqn. S7 reduces to

$$\begin{aligned} S_{detected}\left( \vec{k}_{out} \right)= \int_{\frac{\vec{k}_{in}}{\left| k_{in} \right|}\leq1} S_{in}\left( \vec{k}_{in} \right)\left| G\left( \vec{k}_{out}, \vec{k}_{in} \right) \right|^{2}\left| A\left( \vec{k}_{in}-\vec{k}_{out} \right) \right|^{2}d\vec{k}_{in}\#\left( S10 \right) \end{aligned}$$

Finally, we retrieve Eqn. S1 by substituting the Poynting vector per unit k-space by the photon flux per unit k-space, which is proportional to the Poynting vector.

In the single-pixel imaging configuration, we assume for simplicity that our detector receives light from a narrow acceptance angle range around a nominal angle $\theta=0^{\circ}$ (Fig. 2a) for an integration time $\tau$, such that the number of detected photons $N_{detected}$ is

$$\begin{aligned} N_{detected}=\tau Q_{e}(\Delta k_{D})^{2}L^{2}\int_{\frac{\vec{k}_{in}}{\left| k_{in} \right|}\leq1} I\left( \vec{k}_{in} \right)\left| G\left( \vec{0}, \vec{k}_{in} \right) \right|^{2}\left| A\left( \vec{k}_{in} \right) \right|^{2}d\vec{k}_{in}\#\left( S11 \right) \end{aligned}$$

where $(\Delta k_{D})^{2}$ indicates the k-space area (acquisition angle) of the detector and is constant across all measurements, $Q_{e}$ is the quantum efficiency, the ratio of incident photons to collected charge carriers, and $L^{2}$ denotes the aperture area on which light is incident. The quantum efficiency more generally can be replaced by any measure of detector efficiency. Note that to apply a similar analysis for multi-pixel imaging, Eqn. S1 should be used at multiple different wavevectors $\vec{k}_{out}$.

**2. Accounting for variable losses via normalization**

Consider for simplicity that $G, I$ in Eqn. S11 vary slowly relative to our array factor $A$. In that case, assuming a point-by-point imaging approach, we can model $\left| A \right|^{2}$ as a delta function in space.

Then, the imaging process can be described as follows. At each time, we select a target point $\vec{k}_{t}$ and set the metasurface configuration such that the square of the array factor can be approximated as $\left| A\left( \vec{k}_{in} \right) \right|^{2}={|A}_{\vec{k}_{t}}\left. \right|^{2}\delta\left( \vec{k}_{in}-\vec{k}_{t} \right)$. The quantity ${|A}_{\vec{k}_{t}}\left. \right|^{2}$ is the proportion of coupled power which scatters back into plane waves for measurement of $\vec{k}_{t}$ and accounts for absorption losses in the metasurface. Under this assumption, Eqn. S11 simplifies further to

$$\begin{aligned} N_{detected}[\vec{k}_{t}]=\tau Q_{e}(\Delta k_{D})^{2}L^{2} \eta_{\vec{k}_{t}}I_{in}\left( \vec{k}_{t} \right)\#\left( S12 \right) \end{aligned}$$

The square brackets denote the fact that $N_{detected}$ is not a continuous function of $\vec{k}_{t}$ but rather the number of photons obtained by a discrete measurement. We define the metasurface scattering efficiency $\eta_{\vec{k}_{t}}={|A}_{\vec{k}_{t}}\left. \right|^{2} \left| G\left( \vec{0}, \vec{k}_{t} \right) \right|^{2}$, which is the ratio of incident power from $\vec{k}_{t}$ to scattered power and accounts for both losses due to light failing to couple into the metasurface and absorption losses. This value is generally angle-dependent and depends on metasurface design and has implications on signal-to-noise ratio (SNR). Note that these losses include only coupling and absorption losses and assume a unity quantum efficiency. Critically, light which couples to the metasurface and scatters away from the detector is not considered a loss, since selectively guiding light from non-target angles away from the detector is fundamentally necessary to our imaging system. This notion of efficiency does not, then, express the total proportion of incident light which is translated to a signal, but rather the proportion of light which could be detected.

As desired, we find that the number of detected photons is directly proportional to the incident photon flux from the target point. To properly reconstruct an image, however, we must characterize the efficiency at each measurement and normalize the collected value. This can be experimentally done by taking measurements under known illumination. We call the normalized measurement $N_{ideal}$, formulated as

$$\begin{aligned} N_{ideal}\left[ \vec{k}_{t} \right]=\left( \eta_{\vec{k}_{t}} \right)^{-1}N_{detected}\left[ \vec{k}_{t} \right]\#\left( S13 \right) \end{aligned}$$

In a practical experiment, we would calibrate a device in an environment with known illumination. We refer to SI.15 for our treatment of this normalization in our simulated image recovery.

**3. Impact of voltage discretization on the beam steering / selective coupling ability of a voltage-addressed active metasurface.**

We find that in a perimeter-control addressing configuration, a voltage-addressed active metasurface can be effectively controlled with as few as 2 bits / perimeter voltage. This results in 4 distinct possible voltage levels at each row and column, which in turn allows 7 possible distinct voltage levels across a scatterer which is gated by the difference of a row and column voltage. Generally, for $n$ bits of control over a single voltage, we get $2^{n}$ possible voltage levels, and $2^{n+1}-1$ possible gating voltages (the additional factor of 2 comes from the combinatorial differences between the row and column voltages).

Under the same modeling assumptions made in the rest of the paper, we study the effects of voltage discretization on beam steering. To separate the effects of discretization from those of imperfect amplitude/phase control, we consider the nearly ideal scatterer response from Fig. 3g, in which we have no amplitude variation and nearly ${360}^{\circ}$ of phase control

$$\begin{aligned} \psi\left( v \right)=2\pi\tanh\left( \frac{v}{3} \right), v\in\left[ -6, 6 \right]\#\left( S14 \right) \end{aligned}$$

Our continuous input voltages are selected as described in SI.9, by a fixed initialization and subsequent optimization. We discretize our input voltage such that the resulting set of allowable voltages is evenly spaced and includes the endpoints $-6V, 6V$. Figure S1 shows resulting simulated beam steering performance, under the assumption of an isotropic antenna factor.

We see that while the beam steering performance (directivity) improves substantially from Fig. S1a (where a single bit is used per voltage) to Fig. S1b (where two bits are used per voltage), the improvements from two to three bits (Fig. S1b and Fig. S1c), and from three bits to a fully continuous voltage (Fig. S1c and Fig. S1d) are relatively small. We quantify these improvements by defining a directivity figure of merit (FOM)

$$\begin{aligned} D=\frac{I\left[ u_{x}^{target}, u_{y}^{target} \right]}{\sum I\left[ u_{x}, u_{y} \right]}\#\left( S15 \right) \end{aligned}$$

where $(u_{x}^{target}, u_{y}^{target})$ is the target steering/coupling direction in normalized k-space, and the sum in the denominator is over all k-space points within the light cone. The square brackets denote the discrete nature of the calculation. For the FOM, we calculate the array factor up to some scaling via a Fast Fourier transform with no zero-padding, which sets the number of far field points used in the calculation. For Fig. 3d-g and Fig. S1, however, we increase the resolution to obtain smooth profiles.


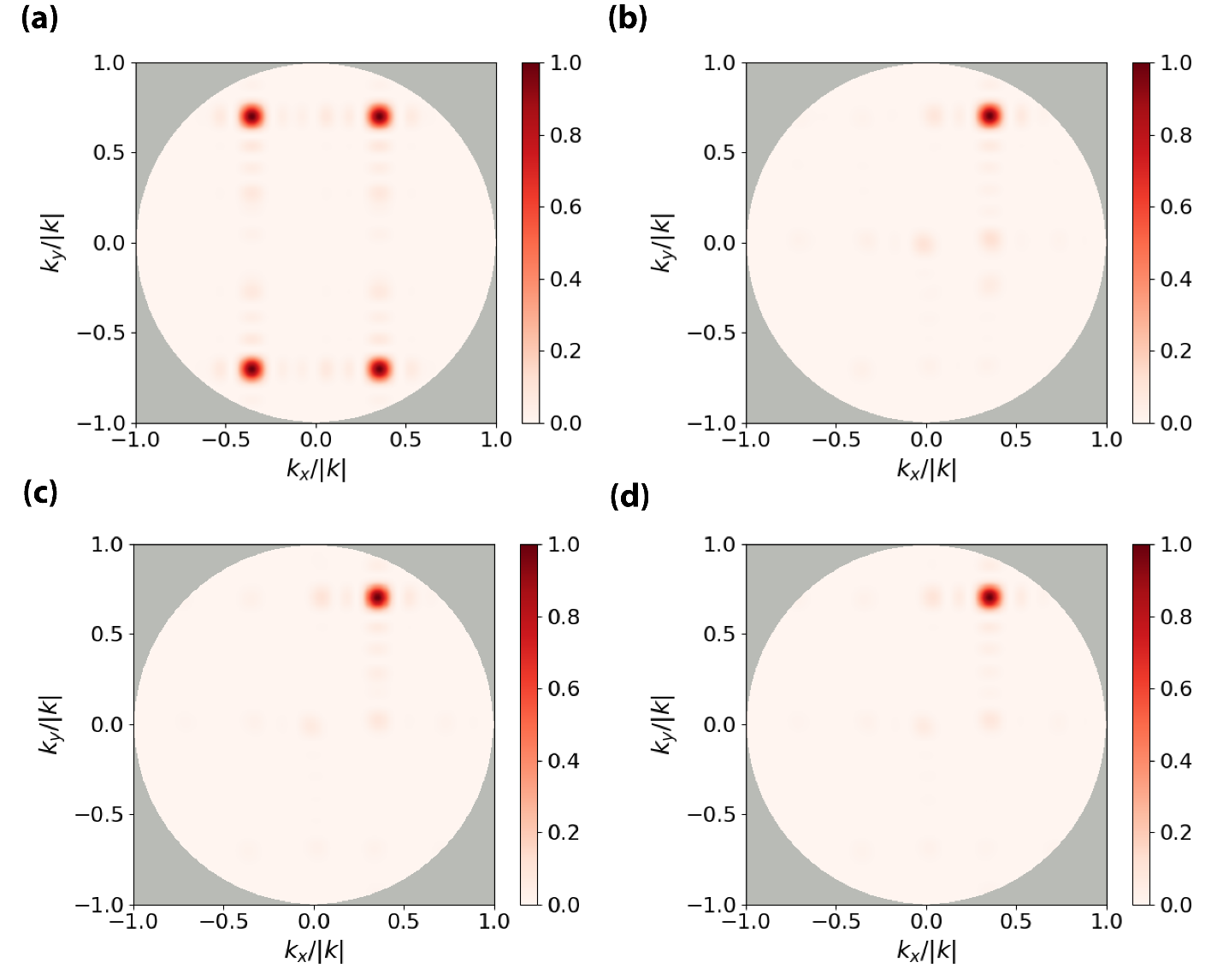


**Figure S1. Effect of voltage discretization on metasurface beam steering performance:** Optimized simulated beam steering performance to $\left( u_{x}, u_{y} \right)=(0.4, 0.75)$, using perimeter-control addressing. The simulation assumes an active metasurface with a $32\times32$scatterers, $13 \mu m\times13 \mu m (8.5\lambda_{0}, 8.5\lambda_{0})$ aperture. We assume isotropic scatterers here. Voltages of each row and column are optimized and then discretized to 1 bit (2 levels per row/column, 3 possible gating voltages) (a), 2 bits (4 levels per row/column, 7 possible gating voltages) (b), 3 bits (8 levels per row/column, 15 possible gating voltages) (c), or not discretized up to machine precision (d).

We find that Fig. S1a-d correspond to $D=0.23, 0.65, 0.72, 0.74$ respectively. This numerically confirms that most of the performance is achieved with only 2 bits per addressing voltage.


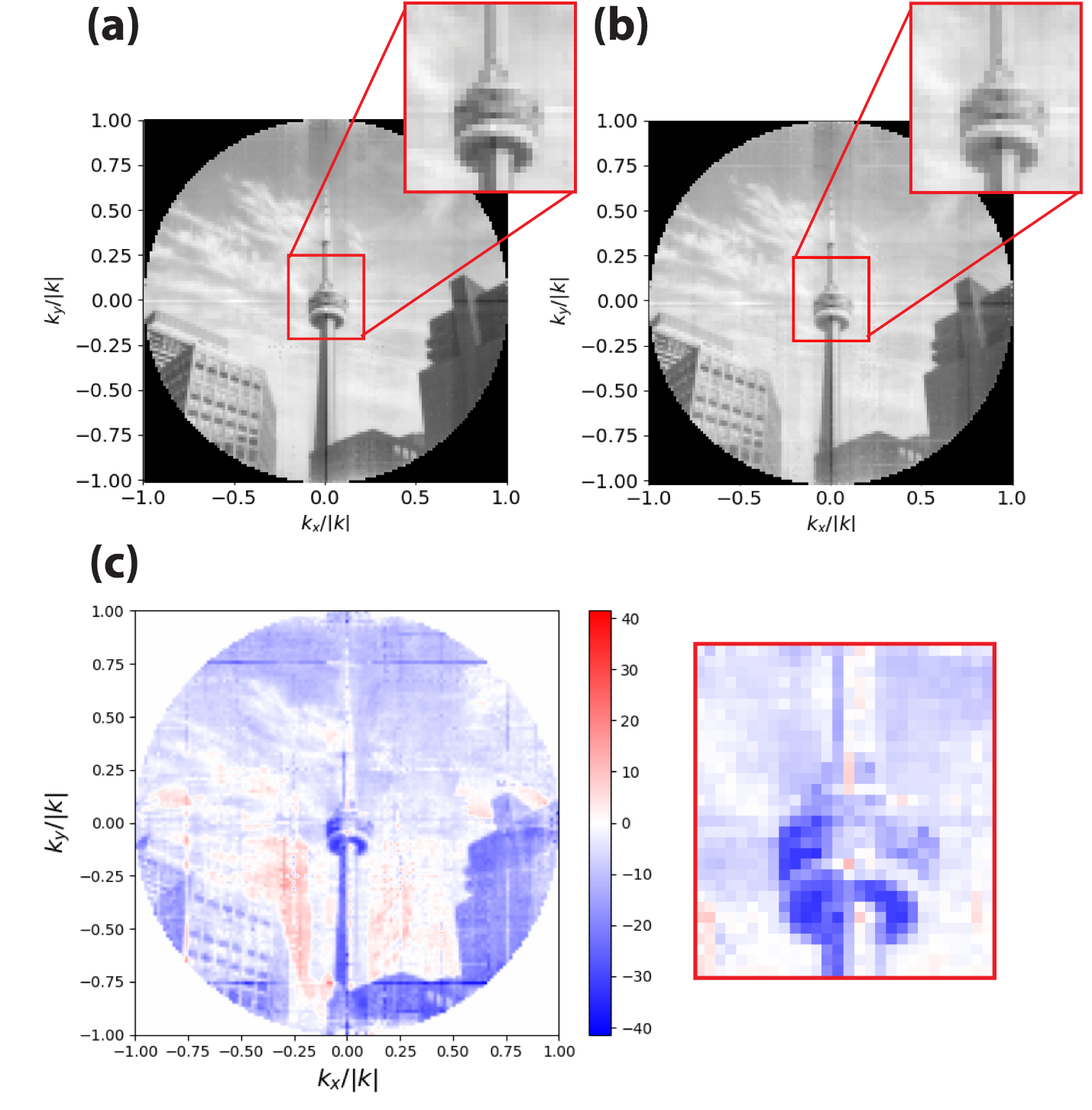


**Figure S2. Effect of voltage discretization on active metasurface image collection:** The simulation assumes an active metasurface with a $255\times255$scatterers, $0.1 mm\times0.1 mm (68\lambda_{0}, 68\lambda_{0})$ perimeter-control addressed aperture. (a) Image recovered with no voltage discretization. (b) Image recovered with each row/column voltage discretized to one of 4 levels (2 bits per voltage). This is equivalent to 7 accessible gating levels. (c) Difference between the normalized images from (a) and (b), with the inset on the right. The maximum difference is $16\%$ of the total range.

We verify that 2 bits per addressing voltage suffices for image recovery in Fig. S2, which treats a larger active metasurface with a $255\times255$ scatterer array. This also demonstrates that our results on discretization apply for larger arrays than demonstrated in Fig. S1. In Fig. S2a, we show an image retrieved with no voltage discretization. We contrast this to Fig. S2b, which is generated from the same set of voltages as Fig. S2a, discretized to 2 bits (4 levels); both figures look very similar, though the image contrast appears stronger in Fig. S2a, and some line artifacts are more pronounced in Fig. S2b (see for example the horizontal lines near the top of the image). We illustrate the difference between the two images in Fig. S2c by normalizing each image to a standard single-channel image range of $[0, 255]$, and subtracting the intensities from Fig. S2b from those of Fig. S2a. Note that the color bar range only spans $\pm40$, across a maximum range of $\pm255$. Thus, we see that the approximation recovered with 2 bits per voltage is sufficient for imaging.

Finally, noting that 2 bits per voltage corresponds to 4 voltage levels per row/column, but 7 possible gating voltages for a scatterer, we extrapolate that 8 possible gating voltages (3 bits) will be sufficient for the beam steering / selective coupling of light from a fully 2D-addressable active metasurface into a single-pixel detector.

**4. Assumed active metasurface characteristics**


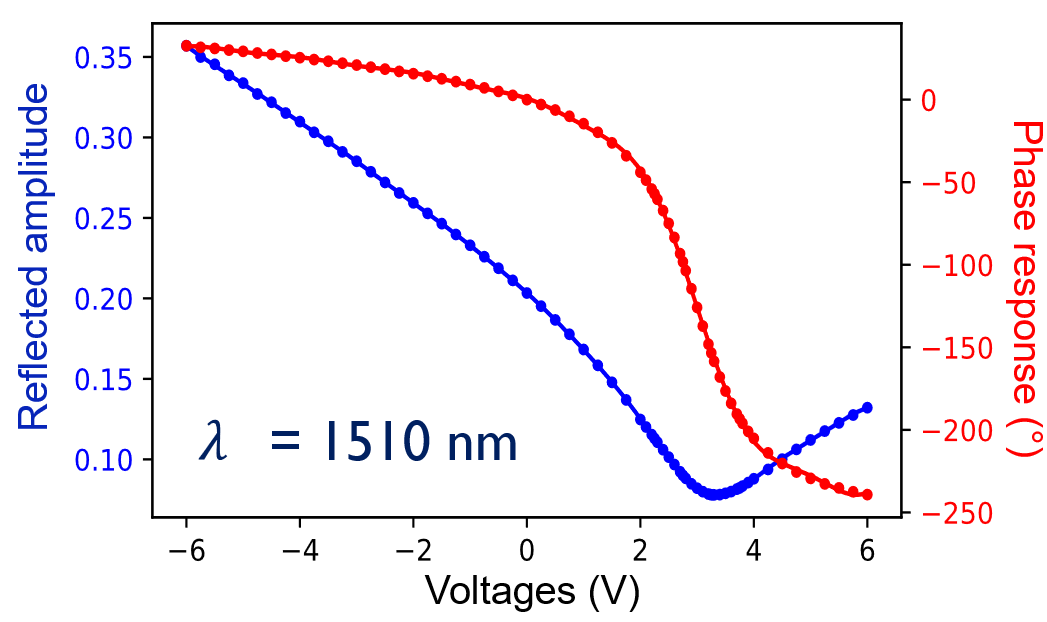


**Figure S3.** Amplitude and phase of scattered light as a function of applied voltage on a TCO-based plasmonic metasurface, at $\lambda_{0}=1509.8 nm$. The dots correspond to data obtained through full-wave simulations and validated against experiment. The lines correspond to a Gaussian RBF best fit.

Our simulation of the ‘realizable’ active metasurface assumes that the amplitude and phase response of individual scatterers follows that of the experimentally demonstrated TCO-based plasmonic metasurface presented in Ref. 2 (see Fig. S3). The dots correspond to data collected from full-wave simulations of the metasurface at $\lambda_{0}=1509.8 nm$, which were found to be in good agreement with experimental performance [2]. The line is the Gaussian Radial Basis Function (RBF) fit used in our simulations. The Gaussian RBF fit is a sum of Gaussian functions, each centered on a voltage data point. We fit the heights of the Gaussians as parameters with Python’s scipy implementation of BFGS and maintain a fixed standard deviation of 1.5 V for each Gaussian.

We additionally assume a dipole coupling (antenna factor) between plane waves and the scatterers in all image collection results, such that the amplitude coupling between a plane wave and the scatterers is $g(k_{x}, k_{y}) =g(\vec{k})\propto\sqrt{1 - {(k}_{y}/|k|)^{2}}$. This antenna factor is not obtained experimentally but rather is selected to highlight image and SNR features which arise from non-isotropic coupling.

**5. The scaling of imaging requirements with metasurface aperture size.**

In this section, we consider how our image acquisition method scales with the number of scatterers along a single axis, $N$. The total number of scatterers is $N^{2}$. We calculate the scaling in general terms through the variables in Table S1, as well as for 2 exemplary cases:

1. A fully 2D reconfigurable aperture of $511\times511$ scatterers, requiring $N^{2}={511}^{2}$ addressing voltages with a discretization of 3 bits/scatterer, with a pitch of $\Gamma=400 nm$, operating at $\lambda= 1509.8 nm$. This is the ‘realizable’ metasurface case which we discuss throughout the work. Generally, these results hold for any metasurface where $\frac{\lambda}{\Gamma}=3.77$.
2. A perimeter-control addressed reconfigurable aperture of $255\times255$ scatterers, requiring $2N=510$ addressing voltages with a discretization of 2 bits/scatterer, considering again the ‘realizable’ metasurface case.

Note that the assumptions on pitch are only used to calculate the number of distinct, useful measurements that can be acquired.

| **Variable** | **Definition** | **Value, case 1 (2D)** | **Value, case 2 (perimeter)** |
| --- | --- | --- | --- |
| $b/s$ | Number of bits per scatterer voltage. | 3 | 2 |
| $N$ | Number of scatterers per row/column. | 511 | 255 |
| $N_{p}$ | Number of distinct points acquired by an image (based on diffraction-limit). | 73,114 | 18,207 |

**Table S1. Metasurface variables for discussion of scaling.**

We calculate the number of image points, $N_{p},$ by assuming a unity numerical aperture (NA) and a linear k-space distance of $FWHM_{1D}$ between points (see SI.6)

$$\begin{aligned} N_{p}\approx\frac{\pi N^{2}}{4H^{2}}\cdot\left( \frac{\Gamma}{\lambda} \right)^{2}, H\approx0.443\#\left( S16 \right) \end{aligned}$$

For our assumed metasurface dimensions, we retrieve Eqn. S17:

$$\begin{aligned} N_{p}\approx0.28N^{2}\#\left( S17 \right) \end{aligned}$$

We additionally consider the following commercially available SRAM [3] in our calculations. Key characteristics are provided in Table S2. This SRAM is only used to provide an order of magnitude for achievable memory and data rates. While it has a large maximum operation bandwidth and amount of memory relative to many other chips, it would generally also be possible to use multiple SRAMs simultaneously to increase the amount of memory and data throughput.

| **Metric** | **Definition** | **Value** |
| --- | --- | --- |
| tRC | The row-cycle time. | 2.7 ns |
| w | The WORD width (number of simultaneously operable data lines). | 72 |
| M | The memory (total amount of bits which can be stored on the SRAM). | 1 Gb |

**Table S2. SRAM variables for discussion of scaling.**

***5.1 Scaling of memory requirements***

*Case 1 (2D addressing of* $511\times511$*scatterers):*

We begin with a naive calculation. The on-chip memory, $m_{2D, 511\times511}$, follows Eqn. S18

$$\begin{aligned} m_{2D, 511\times511}=N^{2}\cdot\left( \frac{b}{s} \right)\cdot N_{p}=57Gb \#\left( S18 \right) \end{aligned}$$

We see that $m_{2D, 511\times511}>M$ by over an order of magnitude. This amount of required memory cannot at this time be kept on a single chip (at least with SRAM).

However, the true amount of required memory is likely significantly less than the value stated above. The operation of directional coupling / beam steering across large apertures typically involves the tiling of a steering ‘supercell,’ which shrinks with increased steering angle. It may be sufficient to store a single supercell per measurement. Nevertheless, we offer the naive value to highlight the improvement afforded by the perimeter-control addressing method.

*Case 2 (perimeter-control addressing of* $255\times255$*scatterers):*

We estimate the on-chip memory costs for perimeter-control addressing, $m_{perim, 255\times255}$, as

$$\begin{aligned} m_{perim, 255\times255}=2N\cdot\left( \frac{b}{s} \right)\cdot N_{p}=19Mb\#\left( S19 \right) \end{aligned}$$

This memory requirement, in contrast, is entirely achievable. If we double the aperture size, the same naive calculation gives a memory requirement of $150Mb$, which remains feasible.

Generally, the naive approach results in the following scaling trends when the full diffraction-limited field is being captured

$$\begin{aligned} m_{2D}\propto N^{4}\#\left( S20a \right) \end{aligned}$$

$$\begin{aligned} m_{perim}\propto N^{3}\#\left( S20b \right) \end{aligned}$$

***5.2 Scaling of digital write-times per measurement***

Note that we use $tRC$ as a proxy for the rate at which we can extract information. The specifications specify a maximum of $640 Gb/s$ throughput, which exceeds our derived value, but under the assumption that the throughput value might include the address data and might indicate peak rather than sustained data transfer rates, we chose the more conservative estimate for this device. We calculate the possible bit write rates as in Eqn. S21 and multiply this rate by the number of bits required to set all the voltages.

$$\begin{aligned} r=\frac{w}{t_{RC}}=27Gb/s\#\left( S21 \right) \end{aligned}$$

*Case 1 (2D addressing of* $511\times511$*scatterers):*

The aperture reconfiguration rate, $R_{2D, 511\times511}$can be approximated as

$$\begin{aligned} R_{2D, 511\times511}=\frac{r}{N^{2}\left( \frac{b}{s} \right)}=34kHz\#\left( S22 \right) \end{aligned}$$

*Case 2 (perimeter-control addressing of* $255\times255$*scatterers):*

$$\begin{aligned} R_{perim, 255\times255}=\frac{r}{2N\left( \frac{b}{s} \right)}=26MHz\#\left( S23 \right) \end{aligned}$$

**6. Analytical bounds on resolution and number of resolvable points in a scene**

In this section, we derive the k-space full width half max (FWHM) achievable by an ideal active metasurface as a measure of achievable image resolution. We also obtain an analytic expression for the number of points resolvable by an active metasurface with subwavelength scatterers. To consider the fundamental resolution limit achievable by local active metasurfaces, we first consider a fully “ideal” metasurface with uniform coupling between any plane wave and the scatterer modes (isotropic antenna factor), no material loss, $2\pi$ phase control, and full 2D addressability of elements.

We study the case of point-by-point imaging to bound system performance. To achieve a diffraction-limited resolution, we set a constant phase gradient such that $\psi_{n}^{x/y}=\Delta\psi_{x/y}n_{x/y}$ where light is collected from target wavevector $\vec{k}_{t}=(k_{tx}, k_{ty})$ by setting phase gradients  $\frac{\Delta\psi_{x}}{\Delta_{x}}=-2\pi k_{tx}, \frac{\Delta\psi_{y}}{\Delta_{y}}=-2\pi k_{ty}$. Here, the quantities $\Delta_{x},\Delta_{y}$ are the metasurface pitches along the x and y directions respectively. Then, the array factor of the system becomes (Eqn. S2)

$$\begin{aligned} A\left( \vec{k}_{in} \right)= \sum_{n_{x}, n_{y}} e^{i\left( \Delta\psi_{x}n_{x}+\Delta\psi_{y}n_{y} \right)}e^{i\left( k_{x}^{in}\Delta x n_{x}+k_{y}^{in}\Delta y n_{y} \right)}\#\left( S24 \right) \end{aligned}$$

For notational simplicity, we define $\gamma_{i}=\Delta\psi_{i}+k_{i}^{in}\Delta_{i}, i\in\{x, y\}$, and write

$$\begin{aligned} A\left( \vec{k}_{in} \right)= \sum_{n_{x}} e^{i\gamma_{x}n_{x}}\sum_{n_{y}} e^{i\gamma_{y}n_{y}}=\left( \frac{1-e^{i\gamma_{x}N_{x}}}{1-e^{i\gamma_{x}}} \right)\left( \frac{1-e^{i\gamma_{y}N_{y}}}{1-e^{i\gamma_{y}}} \right)\#\left( S25 \right) \end{aligned}$$

$$\begin{aligned} \left| A\left( \vec{k}_{in} \right) \right|^{2}=\left( \frac{\cos\left( \gamma_{x}N_{x} \right)-1}{\cos\left( \gamma_{x} \right)-1} \right)\left( \frac{\cos\left( \gamma_{y}N_{y} \right)-1}{\cos\left( \gamma_{y} \right)-1} \right)\#\left( S26 \right) \end{aligned}$$

Next, we assume that $N_{x}, N_{y}\gg1$. This allows us to make two statements:

1. The FWHM of our coupling is dominated by $\left| A\left( \vec{k}_{in} \right) \right|^{2}$rather than $\left| G\left( \vec{0}, \vec{k}_{in} \right) \right|^{2}$.
2. We can Taylor expand $\left( \cos\left( \gamma_{i} \right)-1 \right)^{-1}$ around $\gamma_{i}=0$, as at large $N_{i}$, the FWHM occurs at $\gamma_{i}\ll1$.

$$\begin{aligned} \left| A\left( \vec{k}_{in} \right) \right|^{2}=N_{x}^{2}N_{y}^{2}sinc^{2}\left( \frac{\gamma_{x}N_{x}}{2\pi} \right)sinc^{2}\left( \frac{\gamma_{y}N_{y}}{2\pi} \right), \gamma_{i}\ll1\#\left( S27 \right) \end{aligned}$$

where we define $sinc\left( x \right)=\frac{\sin\left( \pi x \right)}{\pi x}$.

This function has a maximum of $N_{x}^{2}N_{y}^{2}$ at $\gamma_{x}=\gamma_{y}=0$. Thus, the coupling intensity drops by half along each axis when

$$\begin{aligned} sinc\left( H \right)=\frac{1}{\sqrt{2}}, H=\frac{\gamma_{i}N_{i}}{2\pi}\approx0.443\#\left( S28 \right) \end{aligned}$$

Then, our FWHM normalized by $|k|$ can be solved as

$$\begin{aligned} FWHM_{i}=\frac{2H\lambda}{N_{i}\Delta_{i}}\#\left( S29 \right) \end{aligned}$$

Thus, a single resolvable point occupies an area of $FWHM_{x}\cdot FWHM_{y}$ in k-space. Finally, we can divide the total k-space area available to our imaging system by the area of a single resolvable point to find the number of distinct points, $N_{p},$ which can be resolved by the system. This total available area depends on the numerical aperture $NA$, or equivalently on the field of view (FOV), accessible to the metasurface.

$$\begin{aligned} N_{p}= \frac{\pi\cdot NA^{2}}{4H^{2}}\left( \frac{N_{x}\Delta_{x}}{\lambda} \right)\left( \frac{N_{y}\Delta_{y}}{\lambda} \right)\#\left( S30 \right) \end{aligned}$$

**7. Fundamental limits on field of view (FOV) of imaging**

This analysis and discussion follows the work of Kim *et al*. [4]. As in the main text, we assume that the metasurface scatterers are distributed on a rectangular grid. We also assume that the grid is subwavelength, that is, that $\frac{\lambda_{0}}{\Delta_{x}}\geq1,\frac{\lambda_{0}}{\Delta_{y}}\geq1$. There are two types of FOV which we consider in this analysis. Though generally, the regions in k-space where aliasing-free imaging is achievable are not circular (i.e., do not directly correspond to a FOV), we report here on the greatest FOV which falls fully within the aliasing-free region, for simplicity. We refer the reader to Kim *et al.*’s work for further analysis on the shape of alias-free regions and the effect of lattice shape [4].

We first introduce a ‘strong FOV,’ in which light can be coupled into the normal without undesired higher coupling orders existing within the light cone. This means that alias-free imaging can be achieved across this FOV given any antenna factor which is non-zero across the full strong FOV. More precisely, it denotes the FOV corresponding to the largest NA that falls fully within the aliasing-free regime [4].

We can begin our analysis with the same array factor derived in SI.6

$$\begin{aligned} \left| A\left( \vec{k}_{in} \right) \right|^{2}=\left( \frac{\cos\left( \gamma_{x}N_{x} \right)-1}{\cos\left( \gamma_{x} \right)-1} \right)\left( \frac{\cos\left( \gamma_{y}N_{y} \right)-1}{\cos\left( \gamma_{y} \right)-1} \right)\#\left( S31 \right) \end{aligned}$$

This coupling is maximized under the following condition

$$\begin{aligned} \gamma_{x}=\Delta\psi_{x}+k_{x}^{in}\Delta_{x}= 2\pi m_{x} \#\left( S32a \right) \end{aligned}$$

$$\begin{aligned} \gamma_{y}=\Delta\psi_{y}+k_{y}^{in}\Delta_{y}= 2\pi m_{y}\#\left( S32b \right) \end{aligned}$$

where $m_{i}$ are integers corresponding to the diffracted order number along the $i$ axis and we assume that $\Delta\psi_{x}, \Delta\psi_{x}\in[-\pi, \pi)$.

We re-express the condition in terms of the target steering in unit k-space. Let $\left( u_{x}, u_{y} \right)=\frac{\lambda_{0}}{2 \pi}(k_{x}, k_{y})$. Note that to be in the light cone, it must be true that $u_{x}^{2}+u_{y}^{2}\leq1$. Then, we can write the unit k-space points at which diffracted orders appear as

$$\begin{aligned} u_{x}=-\left( \frac{\Delta\psi_{x}}{2\pi}-m_{x} \right)\cdot\frac{\lambda_{0}}{\Delta_{x}}=-u_{x0}+m_{x}\frac{\lambda_{0}}{\Delta_{x}}\#\left( S33a \right) \end{aligned}$$

$$\begin{aligned} u_{y}=-\left( \frac{\Delta\psi_{y}}{2\pi}-m_{y} \right)\cdot\frac{\lambda_{0}}{\Delta_{y}}=-u_{y0}+m_{y}\frac{\lambda_{0}}{\Delta_{y}}\#\left( S33b \right) \end{aligned}$$

where $u_{i0}=\frac{\Delta\psi_{i}}{2\pi}\frac{\lambda_{0}}{\Delta_{i}}$ describe the zeroth order diffraction peak position along axis $i$.

The $m_{x}^{th}, m_{y}^{th}$ diffracted orders can exist in the unit k-space domains defined below

$$\begin{aligned} D_{mx, my}=\left\{ \left( u_{x}, u_{y} \right) \right|\left( u_{xo}-m_{x}\frac{\lambda_{0}}{\Delta_{x}} \right)^{2} +\left( u_{yo}-m_{y}\frac{\lambda_{0}}{\Delta_{y}} \right)^{2}\leq1\}\#\left( S34 \right) \end{aligned}$$

where each domain $D_{mx, my}$ is a circle in unit k-space, with unity radius, and centered at $\left( m_{x}\frac{\lambda_{0}}{\Delta_{x}}, m_{y}\frac{\lambda_{0}}{\Delta_{y}} \right)$. The points in unit k-space which are accessible in the aliasing-free regime are those which fall within the domain $D_{00}$ but not any higher order domain, that is

$$\begin{aligned} D_{AF}=D_{00}-D_{mx, my} \forall m_{x}\neq0 or m_{y}\neq0 \#\left( S35 \right) \end{aligned}$$

It can be shown for a metasurface with scatterers positioned along a rectangular grid that the NA which falls fully within the aliasing-free zone is limited by transverse wave vectors which are fully oriented along the x or y axes [4]. This maximum can be written as

$$\begin{aligned} NA_{s}=u_{x}^{max, AF}=\min\left( \frac{\lambda_{0}}{\Delta_{x}}-1,\frac{\lambda_{0}}{\Delta_{y}}-1 \right)\#\left( S36 \right) \end{aligned}$$

Thus

$$\begin{aligned} FOV_{s}=2\arcsin\left( \min\left( \frac{\lambda_{0}}{\Delta_{x}}-1,\frac{\lambda_{0}}{\Delta_{y}}-1 \right) \right)\#\left( S37 \right) \end{aligned}$$

Whether through mechanical filtering of light or through scatterer design, it is generally possible to prevent the coupling of light incoming from an angle greater than a threshold value. This provides us with a ‘weak FOV’ which falls within the Brillouin zone (BZ), in which the undesired higher orders have greater transverse components than the desired coupling order and can thus be filtered out [4]. To get an upper bound on achievable FOV, we thus assume that each scatterer has a designed antenna factor such that the antenna factor is significant within the BZ, and negligible outside of it. This ‘weak FOV’ then denotes the maximum FOV which would be achievable given such an ideally tailored antenna factor [4]. In the limit of this antenna factor, the higher diffraction orders which appear in the light cone but remain outside of the BZ do not interfere with our measurements. The boundary of the BZ is determined by the condition $\left| \Delta\psi_{x} \right|=\left| \Delta\psi_{y} \right|=\pi$ or equivalently $|u_{i0}|=\frac{\lambda_{0}}{{2\Delta}_{i}}$. We thus define our weak NA and FOV as

$$\begin{aligned} NA_{w}=\min\left( \frac{\lambda_{0}}{2\Delta_{x}},\frac{\lambda_{0}}{2\Delta_{y}} \right) \#\left( S38 \right) \end{aligned}$$

$$\begin{aligned} FOV_{w}=2\arcsin\left( \min\left( \frac{\lambda_{0}}{2\Delta_{x}},\frac{\lambda_{0}}{2\Delta_{y}} \right) \right)\#\left( S39 \right) \end{aligned}$$

The listed bounds reflect the direction in which the imaging system is most limited in FOV. In practice, there are certain directions in which non-aliased measurements could be taken beyond the stated angles [4]. It is also important to note that light collection from within the weak FOV may still suffer from lower efficiency than in the strong FOV. In the SNR discussion in the main text, it is assumed that $\frac{\lambda_{0}}{\Delta x}, \frac{\lambda_{0}}{\Delta y}\geq2$ and thus that every imaged point falls within the strong FOV ($FOV_{s}={180}^{\circ}$). However, the analysis remains applicable across a smaller strong FOV.

**8. Comment on the feasibility of wide FOV imaging**


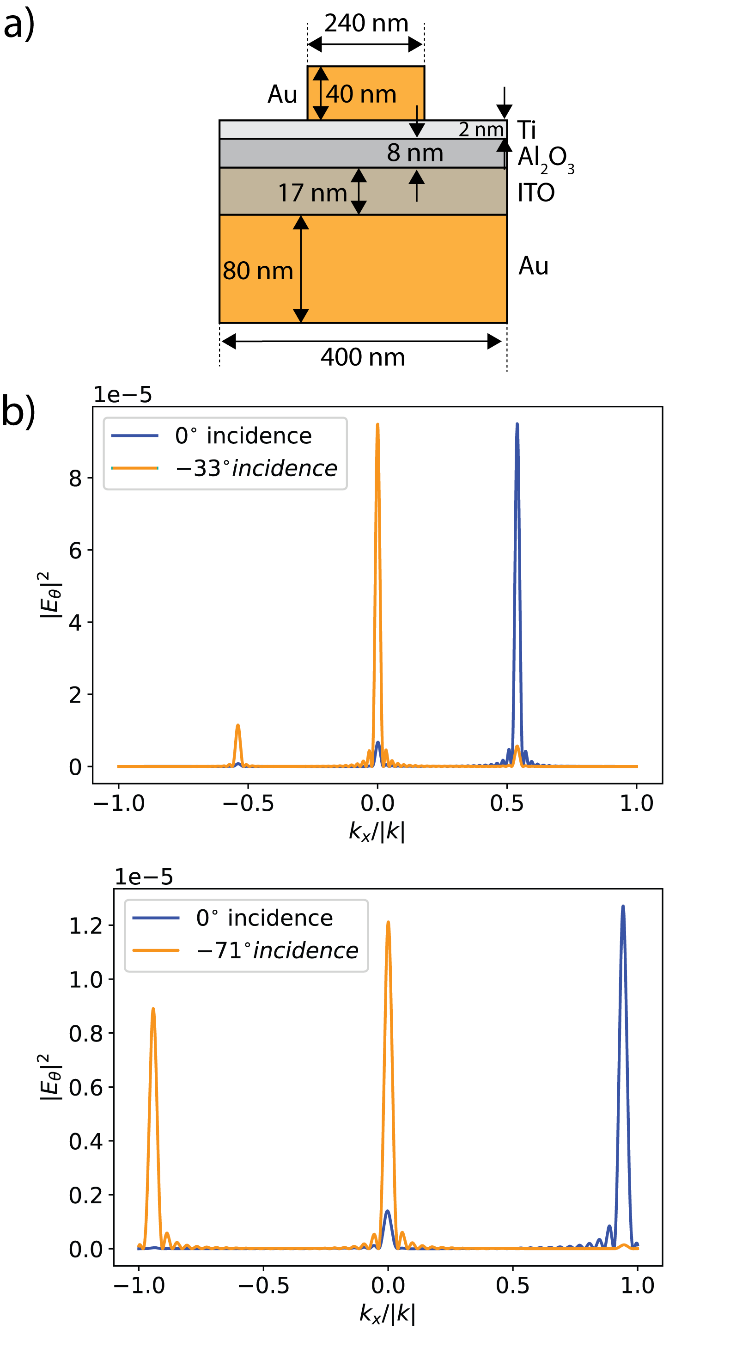


**Figure S4.** FDTD demonstrations of metasurface beam steering reciprocity for $\lambda_{0}=1510 nm$. The metasurface is simulated with periodic boundary conditions around each unit cell.

(a) Simulated scatterer. The total simulated array size consists of 96 of these elements.

(b) Steering simulated with periodic boundary conditions. *Top*: Steering from 7-element unit cell, where the phase difference $\Delta\psi$ between adjacent elements is set to $\Delta\psi=\frac{2\pi}{7}$. This steers the beam to/from a polar angle ${\theta=33}^{\circ}(k_{x}=0.54k)$. *Bottom*: Steering from 4-element unit cell, where the phase difference $\Delta\psi$ between adjacent elements is set to $\Delta\psi=\frac{\pi}{2}$, which steers the beam to/from a polar angle ${\theta=71}^{\circ}(k_{x}=0.94k)$.

In our work, we use the array factor formalism to demonstrate the possibility of wide FOV imaging. In the formalism, it is assumed that the amplitude and phase response of scatterers is constant with the angle of the incoming plane wave, and that said response matches the experimental responses calculated at normal incidence.

While we expect experimental variation in scatterer responses as a function of angle, we note that by reciprocity, our metasurfaces should be able to gather light from a wide angle as efficiently as they can steer light to wide angles. Thus, if we aim to collect light towards normal incidence, it is reasonable to consider the scatterer responses to normal incidence. We verify this intuition through full-wave simulations of a plasmonic stripe antenna metasurface [5] (Fig. S4b) based on Ref. 5, performed with Lumerical FDTD. This simulation confirms that we expect strong beam steering, and conversely light collection, performance up to an angle of ${71}^{\circ}$ with the metasurface design considered (Fig. S4a).

Generally, the metasurface pitch, antenna factor (single scatterer behavior) and inter-element coupling all must be considered when evaluating the FOV over which a metasurface can efficiently collect and steer light. Inter-element coupling can significantly reduce the diffraction efficiency of a metasurface, particularly when steering to wide angles. Our analysis here assumes minimal inter-element coupling, as tends to be appropriate for plasmonic metasurfaces, but we discuss works in which steering was achieved despite significant coupling in the main text [6].

**9. Selection of addressing voltages**

***9.1 Selection of addressing voltages for a fully 2D-addressable aperture***

We select the fully 2D-addressable voltage configuration for each target measurement in a few different ways. For the imaging performance and PSF of an ideal metasurface, we create a blazed grating profile where the phase difference between adjacent elements is $d\psi_{x.y}=-k_{x,y}\Delta_{x, y}$, for scatterers arranged with a period $\Delta_{x, y}$ along $x, y$ and target farfield point $(k_{x}, k_{y})$. For point-by-point imaging with a realizable metasurface, we use a single iteration of the GS algorithm to compute an achievable amplitude/phase at each scatterer, then enforce metasurface properties by the method described in SI.16. We found that this approach generates the same far field coupling pattern as selecting an ideal amplitude/phase according to the blazed grating method and then enforcing the metasurface properties, which we use to generate the realizable PSF data. Finally, we use the GS algorithm to generate our Hadamard and kernel far field couplings, using 25 iterations and multiple initializations to improve the achieved coupling.

***9.2 Selection of addressing voltages for a perimeter-control addressed aperture***

We select the row and column voltages of our perimeter-control addressed metasurfaces for selective angular coupling (beam steering) with two steps: a ‘forward’ design initialization, and a subsequent gradient-based optimization.

For the initialization, we calculate the phase difference between adjacent elements as $d\psi_{x.y}=-2\pi\frac{\Delta_{x, y}}{\lambda}u_{x, y}$. We take an approximate slope of the relationship between applied voltage and resulting phase for our metasurface of choice as $\left( \frac{d\psi}{dV} \right)_{0}$, and calculate the nominal voltage step between adjacent elements as

$$\begin{aligned} dV_{x}= d\psi_{x}/\left( \frac{d\psi}{dV} \right)_{0}\#\left( S40a \right) \end{aligned}$$

$$\begin{aligned} dV_{y}= -d\psi_{y}/\left( \frac{d\psi}{dV} \right)_{0} \#\left( S40b \right) \end{aligned}$$

where the x (row) and y (column) direction have different signs because the column voltages are subtracted from the row voltages. We then set our ‘unwrapped’ voltages, $V_{row}^{u}$ and $V_{col}^{u}$to be sequences of voltages starting at some arbitrary voltage and incrementing with step size $dV_{x}, dV_{y}$. We remap the voltages to ‘wrapped’ voltage sequences, $V_{row}^{w}$ and $V_{col}^{w}$ via a modulo operation

$$\begin{aligned} V_{row,col}^{w}\left[ n \right]=V_{row, col}^{u}\left[ n \right]\%\left( 2V_{max} \right)-V_{max}\#\left( S41 \right) \end{aligned}$$

where $V\in{[-V}_{max}, V_{max}]$ is our range of acceptable voltages (this maximum could be set by power requirements, or by dielectric breakdown conditions).

Finally, because the total gating voltages of our elements the difference between the row and column voltages, we clip the allowed voltages to half the maximum gating voltage at each row/column, such that their maximum difference is limited, and retrieve $V_{row, col}$ as

$$\begin{aligned} V_{row, col}=clip\left( V_{row,col}^{w}, -\frac{V_{max}}{2}, \frac{V_{max}}{2} \right) \#\left( S42 \right) \end{aligned}$$

where the $clip$ operator is an identity function within the bounds given by the second two operators and is set to the closest of the two bounds otherwise.

Once the initialization voltages are selected, we run a constrained optimization with the Python scipy L-BFGS-B optimizer, with optimization bounds $[-\frac{V_{max}}{2}, \frac{V_{max}}{2}]$ on each voltage, on a FOM which is the negative of directivity (Eqn. S15)

$$\begin{aligned} D=-\frac{I\left[ u_{x}^{target}, u_{y}^{target} \right]}{\sum I\left[ u_{x}, u_{y} \right]}\#\left( S43 \right) \end{aligned}$$

where $I$ are intensities computed by the array factor given the input voltages.

**10. Illustrative demonstration of computational post-processing for image recovery**

Figure S5a is reproduced from Fig. 5e-f and shows the point spread function (PSF) of a realizable, fully 2D-addressable array of scatterers. We show the corresponding modulation transfer function (MTF) in Fig. S5b, which is the amplitude of the Fourier Transform of the PSF and indicates the maximum modulation depth of the system as a function of spatial frequency. A larger value for the MTF thus reflects the ability to image high frequency information. As in Fig. S5e-f, we see that while an ‘ideal’ metasurface coupled to a single detector (dashed turquoise line) has the same imaging properties as an ideal pinhole and large sensor array (solid orange line), limited phase response and covariation of amplitude and phase creates aberrations in recovered images (dotted dark blue line).

**
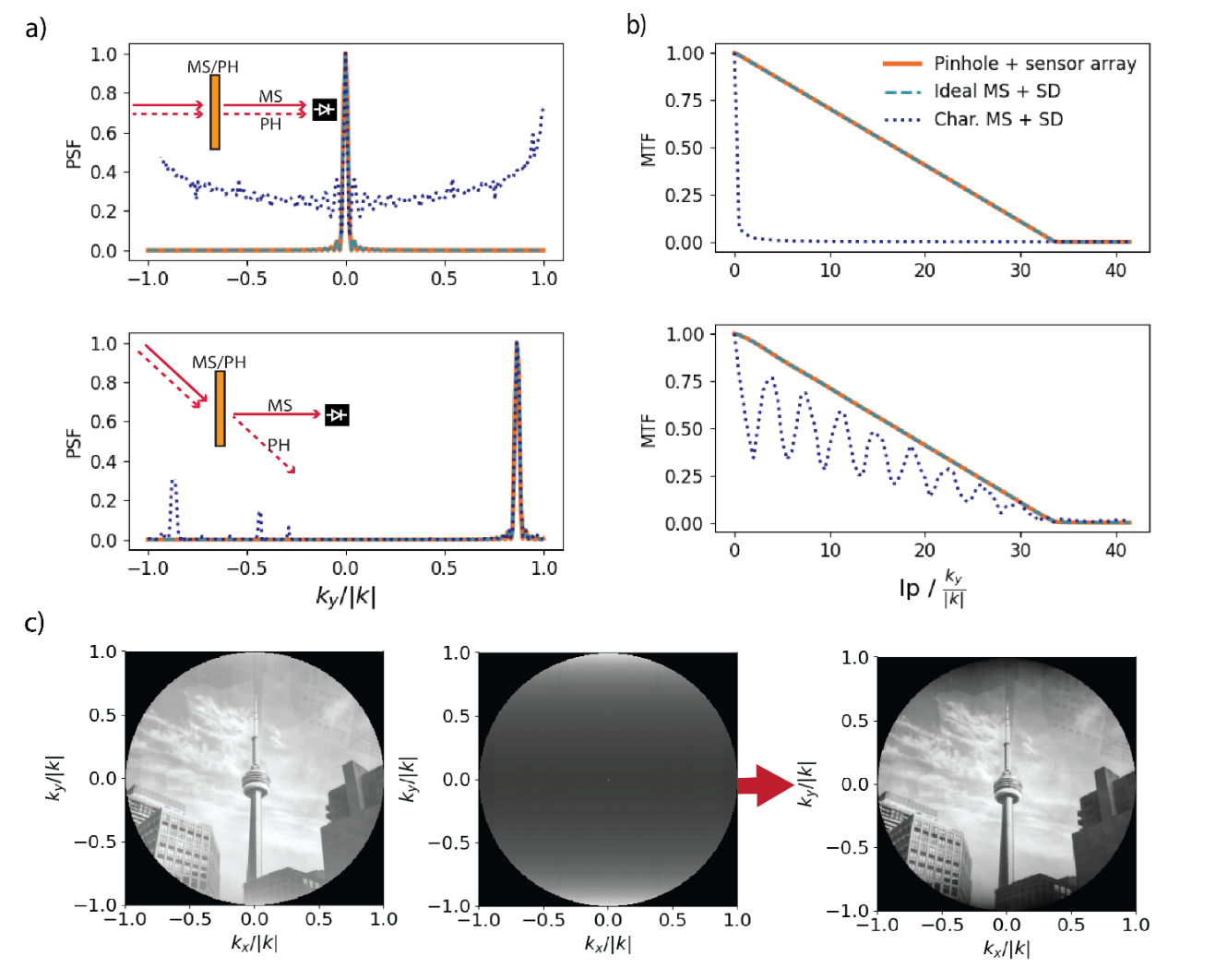
**

**Figure S5.** Imaging characteristics of a diffraction-limited pinhole camera (solid orange line); of an ideal metasurface with isotropic scattering properties, $2\pi$ phase control, and no amplitude variations (dashed turquoise line); and of an experimentally realizable active metasurface single-pixel imaging device of dipole scatterers (dark blue dotted line). The devices simulated in Fig. S5a and S5b are $51 \mu m\times51 \mu m$ ($34\lambda\times34 \lambda)$ in size. (a) Point spread function (PSF) along $k_{x}=0$of a point source originating at $\left( k_{x}, k_{y} \right)=(0, 0)$ (top) and $\left( k_{x}, k_{y} \right)=(0,\frac{\sqrt{3}}{2}|k|)$ (bottom). The insets schematically depict collection of light by an active metasurface (MS) and pinhole (PH). (b) Modulation transfer function (MTF) around the same points as in Fig. S5a. (c) Left: Recovered image from Fig. 5a, showing the effects of the non-idealities of our realizable TCO metasurface. Center: The 2D PSF of the imaging system for a point source at $(0, 0)$. This PSF reflects the different dependency of our antenna factor on $k_{x} and k_{y}$. Right: Post-processed image where the $\left( 0, 0 \right)$ PSF weighted by the $(0, 0)$ intensity is subtracted from the recovered image (left) to improve image contrast.

Because the aberrations do not broaden the PSF but rather introduce predictable, separate peaks, we anticipate that they will be straightforward to correct with computational post-processing. To illustrate this possibility, we consider in Fig. S5c the as-collected image (left) and the two-dimensional $0^{\circ}$ PSF (center) of the device studied in Fig. 5a. Taking the 2D PSF and weighing it by the known intensity of the far field at $0^{\circ}$, we can subtract its contribution from the recovered image. The contrast of the recovered image (Fig. S5c) substantially improves from this simple post-processing step. In practical applications, more sophisticated post-processing could also be used to eliminate the ‘ghost’ image effect discussed in the text, as well as the ‘stripping’ effects which appear in perimeter-control addressed image retrieval. Previous work has also shown that unwanted coupling from the far field can be reduced by further array-level inverse design [7], which would improve the unprocessed PSF—this inverse design approach was used in the text for the perimeter-control addressed images, but not for the fully-2D addressable image.

**11. Derivation of shot noise dominated SNR**

We use the following formulation for the image-wide signal-to-noise ratio (SNR) in Fig. 6 of the main text

$$\begin{aligned} SNR=\frac{\sum_{k_{x}, k_{y}} N_{ideal}\left[ k_{x}, k_{y} \right]^{2}}{\sum_{k_{x}, k_{y}} \eta^{-1}\left[ k_{x}, k_{y} \right]N_{ideal}\left[ k_{x}, k_{y} \right]}\#\left( S44 \right) \end{aligned}$$

where $\eta\left[ k_{x}, k_{y} \right]$ and $N_{ideal}$ are the metasurface scattering efficiency and normalized photon count as discussed in Eqn. S13.

We derive the following from the spatial domain SNR described by Gonzalez and Woods in Chapter 5 of Digital Image Processing [8]

$$\begin{aligned} SNR=\frac{\sum_{k_{x}, k_{y}} S\left[ k_{x}, k_{y} \right]^{2}}{\sum_{k_{x}, k_{y}} E\left[ k_{x}, k_{y} \right]^{2}}\#\left( S45 \right) \end{aligned}$$

where $S$ is the imaged intensity and $E$ is the error in intensity at each measurement. We assume that shot noise dominates the noise in the system, to quantify the impact of the active metasurface on the collected image SNR, noting that more generally we would expect specific detectors to contribute to noise in their own way. The shot noise depends on the number of photons detected, $N_{detected}$, which can be derived from the rate of photon arrival $P (photons/s)$ at the detector, quantum efficiency $Q_{e}$, and measurement integration time $\tau$. Since the standard deviation of shot noise $\sigma_{shot}$ goes like the square root of the average number of events, we can write

$$\begin{aligned} \sigma_{shot}=\sqrt{N_{detected}}\#\left( S46 \right) \end{aligned}$$

This value depends on the far field, the efficiency with which light couples into the metasurface, and the metasurface losses, which vary with the metasurface configuration.

We note that the image is reconstructed from the ideal photon count, $N_{ideal}$, as specified in the manuscript, which is obtained by rescaling the detected photon count.

$$\begin{aligned} N_{ideal}\left[ \vec{k}_{t} \right]=\left( \eta_{\vec{k}_{t}} \right)^{-1}N_{detected}\left[ \vec{k}_{t} \right]\#\left( S47 \right) \end{aligned}$$

Critically, the error introduced by shot noise is also scaled. Then, we can re-express Eqn. S45 as

$$\begin{aligned} SNR=\frac{\sum_{k_{x}, k_{y}} N_{ideal}\left[ k_{x}, k_{y} \right]^{2}}{\sum_{k_{x}, k_{y}} \left( \left( \eta[k_{x}, k_{y}] \right)^{-1}\sqrt{N_{detected}\left[ k_{x}, k_{y} \right]} \right)^{2}}\#\left( S48 \right) \end{aligned}$$

$$\begin{aligned} SNR=\frac{\sum_{k_{x}, k_{y}} N_{ideal}\left[ k_{x}, k_{y} \right]^{2}}{\sum_{k_{x}, k_{y}} \left( \left( \eta[k_{x}, k_{y}] \right)^{-1}\sqrt{\eta[k_{x}, k_{y}]N_{ideal}\left[ \vec{k}_{t} \right]} \right)^{2}}\#\left( S49 \right) \end{aligned}$$

We see that we obtain Eqn. S44 by simplifying the denominator. Finally, we get the SNR in dB as

$$\begin{aligned} SNR_{dB}=10\log_{10} \left( SNR \right)\#\left( S50 \right) \end{aligned}$$

**12. Impact of the k-space detector width (angular acceptance range)**

We consider the impact of detector width, $\Delta k_{D},$ on the resolution of our imaging system through the PSF/MTF and observe two regimes of operation: a diffraction-limited regime $(\Delta k_{D}< FHWM_{min})$ and a detector-limited regime $(\Delta k_{D}> FHWM_{min})$. For both the ideal and realizable metasurfaces, we find that the PSF and MTFs of the imaging system have little dependence on $\Delta k_{D}$ in the diffraction-limited regime where $\Delta k_{D}<FWHM_{min}$ (Fig. S6a, solid lines). However, for $\Delta k_{D}\geq FWHM_{min}$, shown as dotted lines in Fig. S6a, the PSF broadens proportionally to $\Delta k_{D}$, resulting in a diminished ability to resolve higher frequency features (Fig. S6a, MTFs). This behavior is expected, as the system PSF is the convolution of the metasurface PSF and the detection window [9]. Then, the number of resolvable points decreases proportionally to $\frac{1}{\left( \Delta k_{D} \right)^{2}}$ (Fig. S6b).

For a fixed aperture size, however, the rate of photon arrival at the detector increases proportionally to $\left( \Delta k_{D} \right)^{2}$. Thus, an increased detector width can improve the SNR of an image at the expense of resolution. This also indicates that detector coupling width should never be designed to less than $FWHM_{min}$, as it results in a decrease in efficiency with no gain in resolution.

***12.1 Impact of k-space detector width on the image-wide SNR***

In this section, we treat the image-wide SNR as given by Eqn. S44.

Assuming again a shot-noise limited system, we see that the SNR is improved by increased numbers of ideally collected photons and higher metasurface scattering efficiencies. Thus, the shot-noise limited image SNR, shown as black lines in Fig. S6b for different integration times, increases with a wider collection angle. We find that for a fixed imaging time, $SNR\propto\left( \Delta k_{D} \right)^{4}$ since the time available for each measurement is inversely proportional to the number of measurements, and the photon count collected per unit time scales with detector area.

**
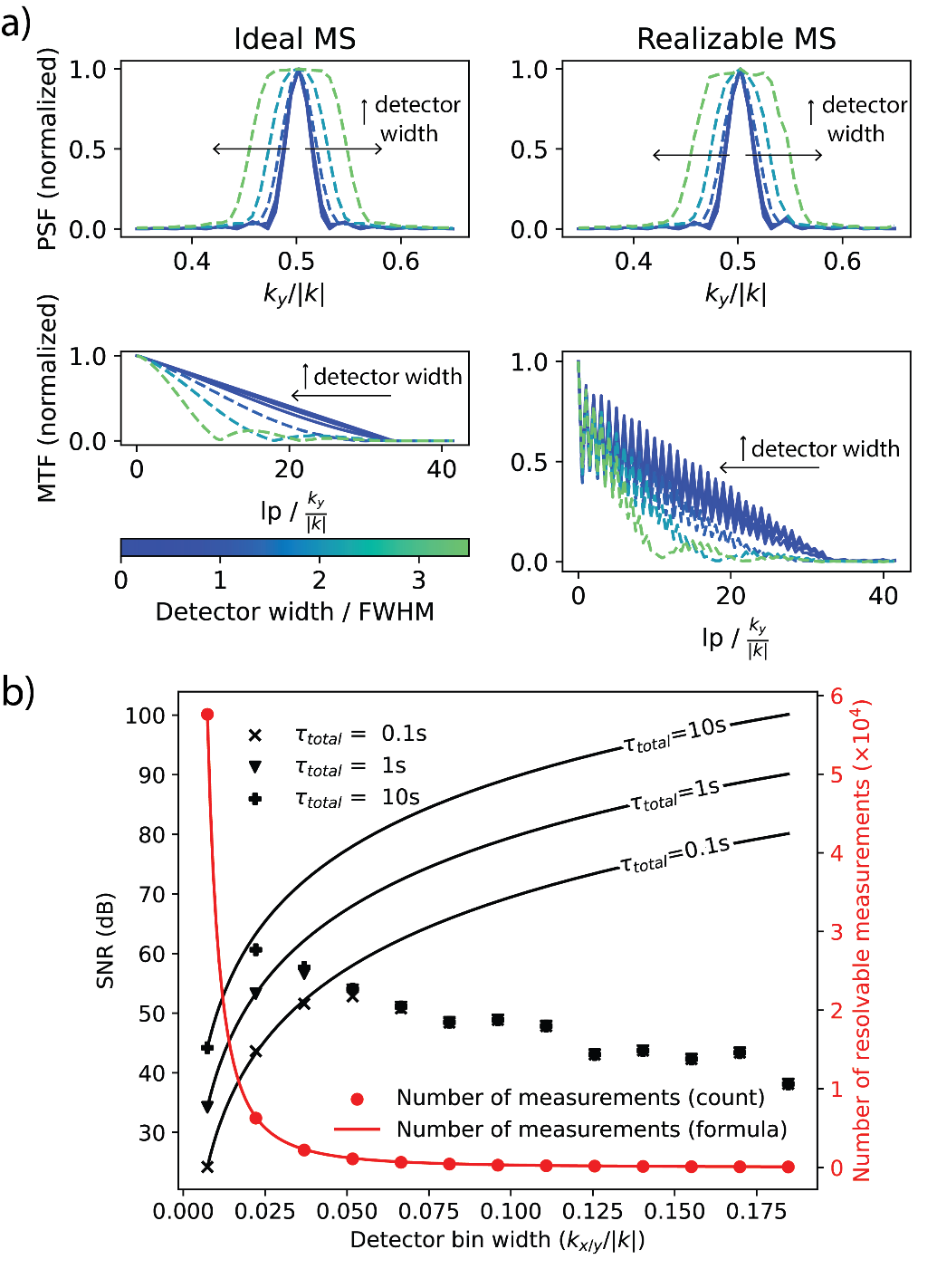
**

**Figure S6.** Effects of detector bin width (acceptance angle) and integration time on SNR of scenes. Results assume a quasi-monochromatic light source with a photon flux of $7.6 \times{10}^{21}\frac{photons}{m^{2} s}$, an aperture size of $0.2 mm\times0.2 mm$, and a single detector with a uniform, square acceptance in k-space. (a) PSF (top) and MTFs (bottom) of ideal (left) and realizable (right) active metasurface single-pixel imager for different detector widths. Solid lines indicate diffraction-limited resolution, whereas dotted lines show detector-limited resolutions. (b) SNR in dB (black, left axis) and number of distinguishable image points (red, right axis) as a function of detector width. The black lines are the shot-noise limited SNR. The black points are the SNR with both shot noise and scene-dependent normalization errors.

To simplify our analysis, we assume a square detector in k-space with a width of $\Delta k_{D}$ such that the detection efficiency $D$ as a function of wavevector$\left( k_{x},k_{y} \right)$ is

$$\begin{aligned} D\left( k_{x},k_{y} \right)=Q_{e} \Theta\left( \frac{\Delta k_{D}}{2}-\left| k_{x} \right| \right)\Theta\left( \frac{\Delta k_{D}}{2}-\left| k_{y} \right| \right) \#\left( S51 \right) \end{aligned}$$

where $\Theta$ is the Heaviside step function and $Q_{e}$ is the quantum efficiency, the ratio of incident photons to collected charge carriers. We generally expect that the trends observed in Fig. S6a of the manuscript will hold for other detector shapes as well.

We explicitly calculate the number of resolvable points by dividing the total k-space by the width of a detection bin in k-space. In Fig. S6b, this calculation assumes that we can image a FOV of ${180}^{\circ}$. The red line is computed directly from the ratio of areas. The red dots are a count of the number of measurements which fully fit our FOV and consider the fact that as bin width increases, discretization effects make the number of available measurements differ from the ratio of areas.

However, in addition to reducing the resolution, increased detector width may introduce a far field dependent normalization error in cases where the antenna factor amplitude is not isotropic. This error arises because we cannot in this case determine the exact coupling loss associated with measurements and is further explained in SI.17.

The effect of the normalization error on our realizable system is included alongside the shot noise error in the scatterplots of Fig. S6b. Shot noise dominates for small detector widths, but as the detector width increases, the normalization error becomes more significant, limiting the SNR to ~40 dB with our chosen bin sizes and the angular dependency of our coupling. This normalization error will decrease to zero as the coupling becomes isotropic over the FOV of interest.

***12.2 Impact of k-space detector width on single-measurement SNR***

Consider a total image acquisition time $\tau$, and a fixed aperture size. Here, we define the single-measurement SNR, $SNR_{sp},$ as

$$\begin{aligned} SNR_{sp}=\frac{N}{\sigma_{N}}\#\left( S52 \right) \end{aligned}$$

where $N$ is the expected number of photons (signal power) and $\sigma_{N}$ is the expected noise power (expressed as the standard deviation of the signal). We assume again a shot noise limited system where $\sigma_{N}=\sqrt{N}$ such that

$$\begin{aligned} SNR_{sp}=\sqrt{N}\#\left( S53 \right) \end{aligned}$$

We first consider a lensless active metasurface single-pixel imager. The number of image points which we can collect, $N_{p}$, follows

$$\begin{aligned} N_{p}\propto\frac{1}{\left( \Delta k_{d} \right)^{2}}\#\left( S54 \right) \end{aligned}$$

Then, the acquisition time per measurement of a point in space, $\tau_{p}$, scales like

$$\begin{aligned} \tau_{p}=\frac{\tau}{N_{p}}\propto\left( \Delta k_{d} \right)^{2}\#\left( S55 \right) \end{aligned}$$

The rate of photons acquisition, $P$, is also proportional to the acquisition k-space area

$$\begin{aligned} P\propto\left( \Delta k_{d} \right)^{2}\#\left( S56 \right) \end{aligned}$$

Finally, the total number of acquired photons in a measurement is

$$\begin{aligned} N=P\tau_{p}\propto\left( \Delta k_{d} \right)^{4}\#\left( S57 \right) \end{aligned}$$

Thus, the shot noise limited SNR follows trend

$$\begin{aligned} SNR_{sp}\propto\left( \Delta k_{d} \right)^{2}\propto\frac{1}{N_{p}}\#\left( S58 \right) \end{aligned}$$

We can also consider the per-measurement (per-pixel) SNR of a conventional lens-coupled sensor array, $SNR_{lc}$, assuming the same acquisition time $\tau$, fixed aperture size, and shot noise limited operation. Thus, again, $SNR_{lc}=\sqrt{N}$. However, the number of photons collected is different. We replace the notion of k-space detector width, $\Delta k_{d}$, by a pixel pitch, $\gamma_{p}$. In that case, the per-pixel rate of photon acquisition, $P$, scales like the pixel area and thus like the inverse of the pixel count

$$\begin{aligned} P\propto\gamma_{p}^{2}\propto\frac{1}{N_{p}}\#\left( S59 \right) \end{aligned}$$

The total number of photons acquired per pixel is

$$\begin{aligned} N=P\tau\propto\frac{1}{N_{p}}\#\left( S60 \right) \end{aligned}$$

and the shot noise limited SNR scales like

$$\begin{aligned} SNR_{lc}\propto\frac{1}{\sqrt{N_{p}}}\#\left( S61 \right) \end{aligned}$$

We see that for a large number of measurements, the scaling of SNR is more advantageous for conventional lens-coupled sensor arrays.

**13. Single-pixel edge detection and computational edge detection**

We use the reconfigurable array factor of the active metasurface to extract the location of edges in the scene at low computational cost. A fully 2D addressable metasurface is assumed to retrieve these results. We focus on edge-detection due to its significance in machine vision applications [10] and use Fig. S7a as our choice of scene because it has distinct edges of varying scale and orientations. The full image covers a ${180}^{\circ}$ FOV, however, we aim to detect edges only out to the shaded line (${128}^{\circ}$ FOV) because we need spatial information around a point to detect an edge, which we cannot have at the boundary of the scene.

Edges of an image are commonly found by the computational convolution of an initial image and various kernels, which can be computationally expensive on large images. Here, we instead perform our convolutions by selecting metasurface configurations which weigh the coupling of light into the detector by edge-detection kernels. We find that this method allows us to perform various types of edge detection (Fig. S7b-d).

To properly compare our proposed single-pixel edge detection approach to common methods of computational edge detection, it is useful to consider a standard workflow for edge detection (Fig. S8b). In computational edge detection, an initial image is acquired and converted to a digital array of intensities. It may also be downscaled, to speed up the edge detection process at the expense of some loss of information. The image is then blurred, enough to reduce noise but not so much that it eliminates relevant features, and convolved with edge detection kernels. In each convolution, an $M\times M$ kernel matrix is pointwise multiplied with an $M\times M$ block of our $N\times N$ image. The image block being multiplied with the kernel matrix is shifted across the full image with some stride $S$ (i.e., the pointwise matrix multiplication is taken every $S$ pixels). Finally, information from different kernels is recombined as necessary, here by a sum of squares and thresholding. The overall runtime of the convolution is $O(N^{2}M^{2}/S^{2})$.

**
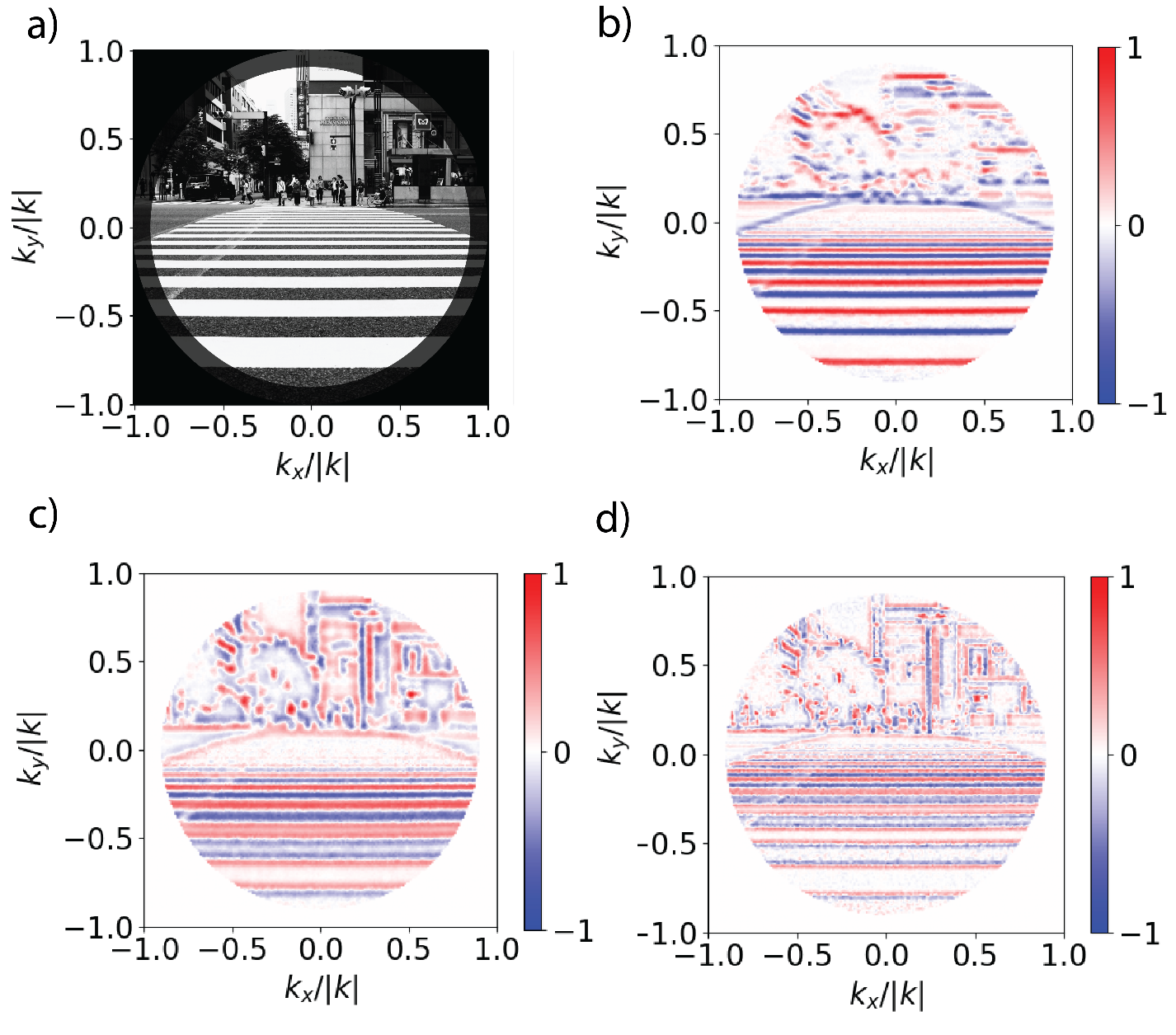
**

**Figure S7.** Simulated single-pixel edge-detection via an active metasurface imaging device. All results were generated assuming an active metasurface with $345\times345$ scatterers, $0.14 mm\times0.14 mm$ ($91 \lambda_{0}\times91 \lambda_{0})$.

(a) Quasi-monochromatic (single wavelength) ground truth scene. The bright circle depicts a 128° FOV and is the part of the scene over which edge detection is performed. The dimmer circle extends to a 180° FOV. Picture by Daryan Shamkhali on Unsplash.

(b-d) Simulated edges of the ground truth scene detected by each type of optimized kernel. Direction-sensitive edge detection with a Sobel kernel (b), edge detection of large features with a large Laplacian of Gaussian kernel (c), edge detection of small features with a small Laplacian of Gaussian kernel (d).

For large images and kernel sizes, this process becomes computationally expensive due to the repeated pointwise multiplication of matrix elements. For this reason, it is common for images to be downscaled and for kernel sizes to be limited to 3 x 3 or 5 x 5 pixels, though in recent years there has been renewed interest in large kernel designs in convolution neural networks (CNNs) [11]. To identify larger image features with small kernels, it becomes necessary to either downscale the initial image until image features are on the scale of ~5 x 5 pixels, or more commonly, to build in sequential layers of convolution as in CNNs. In Fig. S8b, we choose to use 7 x 7 Sobel kernels for edge detection, though in CNNs, kernels are often trained and optimized rather than pre-determined.

Active metasurfaces offer an alternate way of taking the convolution of an image with a kernel, compatible with single-pixel detection. We can obtain the convolution of the scene with an arbitrary kernel at a point by setting the array factor to first the positive, then the negative component of said kernel, centered at each point of interest. The first measurement then gives us the convolution of the positive portion of our edge-detection kernel with the scene, up to some known rescaling, and the second measurement does the same for the negative portion of our edge-detection kernel. The scalar difference of these two measurements gives us the measurement at the point, without requiring pointwise multiplication of matrices. We show the results of this process in Fig. S8d (duplicated from Fig. S7b-d). As in our Hadamard image reconstruction, we note that imperfect kernels (Fig. S8c) nevertheless result in clear edge detection. From left to right, we demonstrate directional (Sobel) edge detection, large-feature edge detection (large Laplace of Gaussian), and small-feature edge detection (small Laplace of Gaussian) to showcase the versatility of the approach. In these figures, we evaluate the kernels at 21 265 distinct points (42 530 measurements), but we could reduce the number of measurements by increasing the stride (spacing) between adjacent measurements as is often done in computational edge-detection.

Thus, to perform edge detection, we can either acquire a full image to a desired resolution and go through the computational steps described above, with a relatively computationally expensive pointwise matrix multiplication step associated with each image point, or at most double the number of measurements taken (depending on choice of stride) and take the difference of two scalars at the same image points. We expect this second approach to be particularly valuable for spatially large convolution kernels, as the acquisition time can be decoupled from choice of kernel (though losses may vary in a practical system), unlike the computational cost of the matrix multiplication. More generally, these results reinforce that the variety of measurements which can be achieved with single-pixel active metasurface devices allows for information to be extracted from the scene without full scene reconstruction. We would recommend that future studies look further into potential applications of the technology in compressive classification and in the design of “smashed” (dimensionally reduced) filters which collect only necessary information for classification [12].


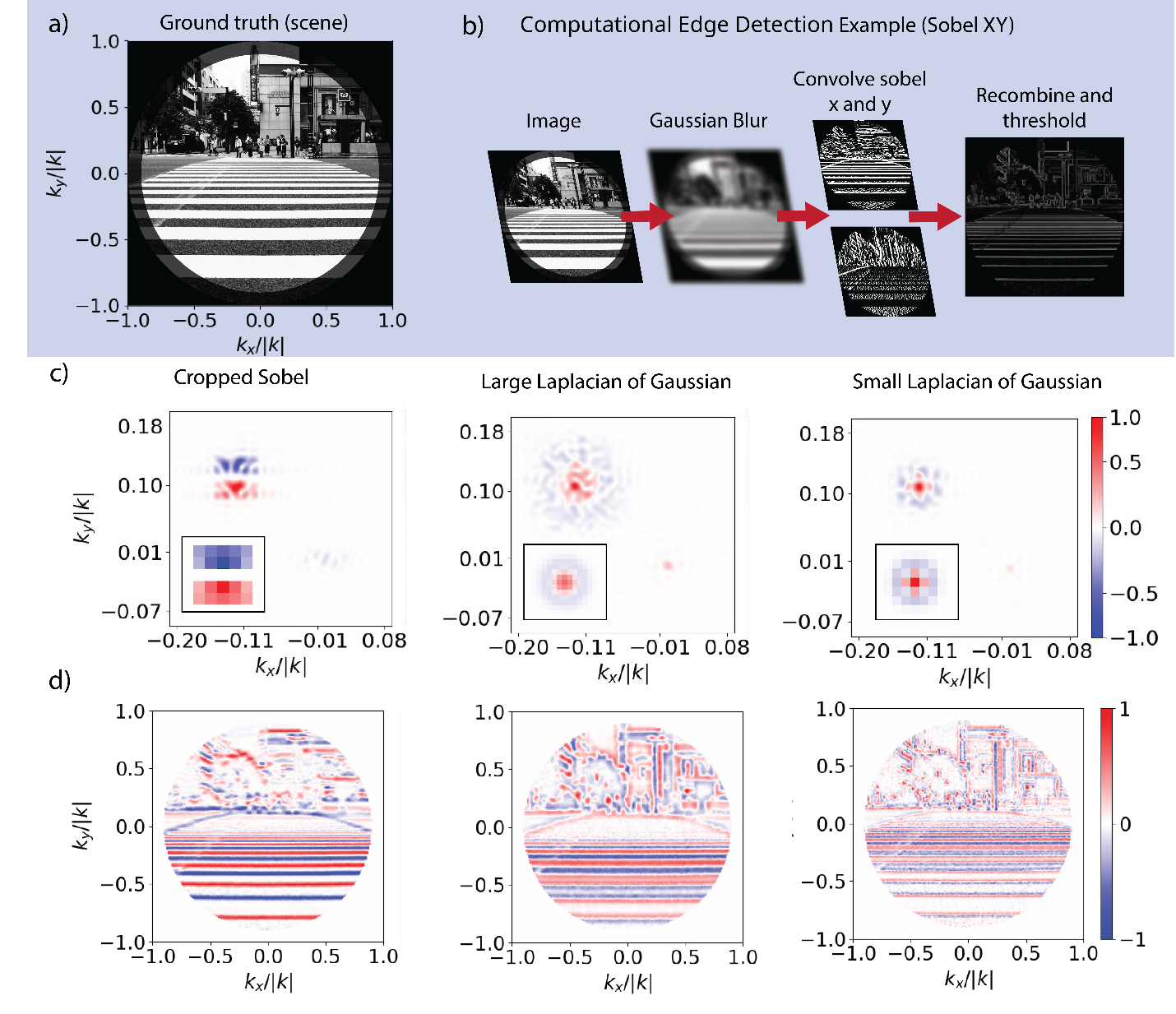


**Figure S8.** Simulated single-pixel edge-detection via an active metasurface imaging device. All results were generated assuming an active metasurface with $345\times345$ scatterers, $0.14mm\times0.14 mm$ ($91 \lambda_{0}\times91 \lambda_{0})$.

(a) Quasi-monochromatic (single wavelength) ground truth scene, replicated from Fig. S7a. The bright circle depicts a 128° FOV and is the part of the scene over which edge detection is performed. The dimmer circle extends to a 180° FOV. Picture by Daryan Shamkhali on Unsplash.

(b) A standard workflow for computational edge detection in machine vision applications.

(c) Map of computed coupling intensities as a function of $k_{x}, k_{y}$ for a pair of measurements. The difference between the two measurements is the product of the far field scene with the coupling. The Sobel kernel (left) approximates a first derivative in $k_{y}$. The Laplacian of Gaussians approximate second order derivatives at a large (center) and small (right) scale. Insets represent the target kernels provided to the G-S optimization.

(d) Simulated edges of the ground truth scene detected by each type of optimized kernel, replicated from Fig. S7b-d. Left to right: Direction-sensitive edge detection, edge detection of large features, edge detection of small features.

**14. Image reconstruction algorithms**

We describe the reconstruction algorithms in terms of ideal photon count, $N_{ideal}$. The procedure by which the ideal photon count is found from the detected photon count $N_{detected}$ is presented in SI.2 and SI.15.

In all imaging simulations, we simulate the far field coupling and far field scene at a higher resolution than the diffraction-limited resolution of the active metasurface. This allows us to treat our measurement results as continuous.

**Point-by-point imaging.** Each measurement is assumed to come from a distinct wavevector in the far field. Then, each wavevector corresponds to a distinct point in space. We generate our image $I[k_{x}, k_{y}]$ as $I\left[ k_{x}, k_{y} \right]=N_{ideal}[k_{x}, k_{y}]$ (up to normalization of the full image).

**Hadamard imaging.** The Hadamard basis is defined as a basis in k-space consisting of a $2^{N}\times2^{N}$ element grid (for integer $N$), where each square can take on a value of either $+1$ or $-1$ in each basis element and all elements are orthogonal. Thus, retrieving the inner product between each basis element and the scene requires two measurements—one for the positive component and one for the negative component. Alternatively, one could take only the positive measurements and calculate the negative measurement from the first basis element (where each grid element is $+1$), halving the total number of required measurements. We opt for the first option in Fig. 7d of the manuscript.

The inner product of the scene with the first basis element is obtained in a single measurement, as that element is positive. For all other elements, we determine the weight attributed to a basis element $i$from the positive and negative measurements $N_{ideal}^{p, i}, N_{ideal}^{n, i}$ as:

$$\begin{aligned} w_{i}=N_{ideal}^{p, i}-N_{ideal}^{n, i}\#\left( S62 \right) \end{aligned}$$

Note that in time-critical applications, measurements should be taken in order of increasing spatial frequency [13].

**Edge-detection/kernel imaging.** We perform edge detection by taking a convolution between an edge-detection kernel and the scene. We understand this as taking the inner product between the far field and an optimized kernel, centering the kernel at each point of the scene. The inner product gives us the strength of an edge at a given point.

As with Hadamard imaging, we take two measurements to resolve the inner product at each point (one for the positive component of the kernel and another for the negative component). In the most general case where the positive and negative parts of the kernel have different weights, we must rescale $N_{ideal}^{p, i}$, $N_{ideal}^{n, i}$ before taking their difference. This is because the physical ideal measurement we take will give the same total weight to positive and negative coupling.

Assume that in our mathematically ideal kernel $K$, the ratio of the positive and negative coupling is

$$\begin{aligned} R_{coupling}=\frac{I_{pos}}{I_{neg}}=\frac{\int\max\left( K\left[ k_{x}, k_{y} \right], 0 \right)d\vec{k}}{\int\max\left( -K\left[ k_{x}, k_{y} \right], 0 \right)d\vec{k}}\#\left( S63 \right) \end{aligned}$$

We then express the strength of an edge centered at $k_{x}, k_{y}$ as

$$\begin{aligned} w_{edge}\left[ k_{x}, k_{y} \right]=N_{ideal}^{p, i}{\left[ k_{x}, k_{y} \right]I}_{pos}-N_{ideal}^{n, i}{\left[ k_{x}, k_{y} \right]I}_{neg}\#\left( S64 \right) \end{aligned}$$

**15. Normalization and the array factor calculation**

As discussed in the manuscript, it is important in image retrieval to normalize the detected photon by the measurement efficiency. In our simulation, this normalization is performed by fixing the following condition to be true

$$\begin{aligned} \int_{\frac{\vec{k}_{in}}{\left| k_{in} \right|}\leq1} \left| G\left( \vec{0}, \vec{k}_{in} \right) \right|^{2}\left| A\left( \vec{k}_{in} \right) \right|^{2}d\vec{k}_{in}=1\#\left( S65 \right) \end{aligned}$$

This section justifies this normalization.

Assume that all light incident on the active metasurface couples to the scatterers and moreover assume that there are no material losses in the resonator. This is the condition which gives us the ideally retrieved photon count which we need for image reconstruction.

We consider first the case where a single wave of unit power is normally incident on the metasurface, such that $I_{in}\left( \vec{k}_{in} \right)=\delta(\vec{k}_{in})$. From Eqn. S1, we determine that

$$\begin{aligned} I_{detected}\left( \vec{k}_{out} \right)=\left| G\left( \vec{k}_{out}, \vec{0} \right) \right|^{2}\left| A\left( -\vec{k}_{out} \right) \right|^{2}\#\left( S66 \right) \end{aligned}$$

Since we assume full coupling and no material losses, it follows that the total scatterer photon flux is equal to the incident photon flux. Thus,

$$\begin{aligned} 1=\int_{\frac{\vec{k}_{out}}{\left| k_{out} \right|}\leq1} I_{detected}\left( \vec{k}_{out} \right)d\vec{k}_{out}=\int_{\frac{\vec{k}_{out}}{\left| k_{out} \right|}\leq1} \left| G\left( \vec{k}_{out}, \vec{0} \right) \right|^{2}\left| A\left( -\vec{k}_{out} \right) \right|^{2}d\vec{k}_{out}\#\left( S67 \right) \end{aligned}$$

By reciprocity, we know that $\left| G\left( \vec{k}_{out}, \vec{0} \right) \right|^{2}=\left| G\left( \vec{0}, \vec{k}_{out} \right) \right|^{2}$. Additionally, we note that the array factor $\left| A\left( \vec{k} \right) \right|^{2}=R\left| A\left( -\vec{k} \right) \right|^{2}$ where $R$ denotes a reflection $k_{x}\to-k_{x}, k_{y}\to-k_{y}$. Then, since our domain of integration is invariant to the reflection, we can write

$$\begin{aligned} 1=\int_{\frac{\vec{k}_{out}}{\left| k_{out} \right|}\leq1} \left| G\left( \vec{0}, \vec{k}_{out} \right) \right|^{2}\left| A\left( \vec{k}_{out} \right) \right|^{2}d\vec{k}_{out}=\int_{\frac{\vec{k}_{in}}{\left| k_{in} \right|}\leq1} \left| G\left( \vec{0}, \vec{k}_{in} \right) \right|^{2}\left| A\left( \vec{k}_{in} \right) \right|^{2}d\vec{k}_{in}\#\left( S68 \right) \end{aligned}$$

Thus, we can use this normalization to find our ideal photon count, $N_{ideal}$.

**16. Gerchberg-Saxton (GS) optimization and enforcing metasurface properties**

The metasurface properties are enforced by expressing the scatterers’ amplitudes/phases given by the GS algorithm as points in the complex plane, and then casting each scatterer’s response to the nearest physically achievable point in the complex plane, as defined by the L2 norm. The casting of arbitrary responses to their nearest achievable points generally depends on the choice of normalization for the scatterer amplitudes (the GS algorithm provides us with the relative but not absolute amplitudes). We choose to normalize the amplitudes of the GS algorithm such that the greatest amplitude is 0.37. This provided us with best convergence. An example of a converged set of points (blue) and their nearest achievable values (red) is provided in Fig. S9.

In the case of the Hadamard basis optimization, we found that the GS algorithm with enforced metasurface properties led to a large, undesirable coupling peak at $\left( k_{x}, k_{y} \right)=(0, 0)$. This coupling peak was reduced by adding a random perturbation to the designed voltages. The results shown in Fig. 7d and Fig. 7e of the manuscript were generated with a voltage perturbation uniformly distributed between -0.9 and 0.9V.


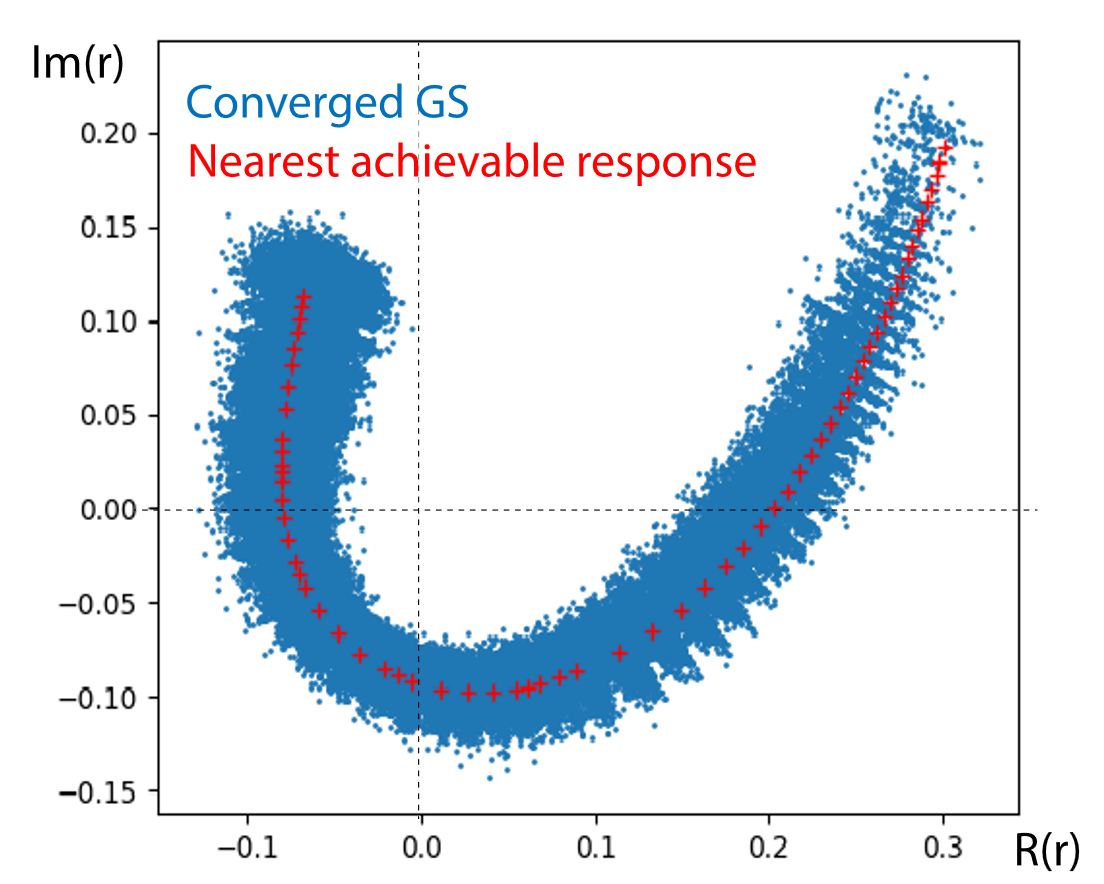


**Figure S9.** Complex response of ${511}^{2}$ individual scatterers as designed by the GS algorithm (blue) and achievable complex response (red) which was used in simulating metasurface performance.

**17. SNR calculations with varying bin widths**

Figure S6b accounts for two sources of errors—the shot noise, as described in Eqn. S44 and a systematic error due to normalization. This error can best be understood through an example. Suppose that an active metasurface has a dipole antenna factor, that the metasurface configuration is set such that each scatterer has the same amplitude and phase response, and that we have a relatively wide detector width $\Delta k_{D}/|k|=0.2$. Assume also for simplicity that the scattering efficiency is only lowered by imperfect coupling at wider angles. Then, the detector will collect light from the normal, $\left( k_{x}, k_{y} \right)=(0, 0)$, with an efficiency of $\eta=1$, as well as from other angles near the normal, including $\left( k_{x}, k_{y} \right)=(0, 0.1)$ with an efficiency of $\eta=0.99$. Not knowing *a priori* where the light is coming from, we cannot distinguish in our measurement the effect of $0.99N$ photons arriving from the normal from $N$ photons arriving from $(k_{x}, k_{y})=(0, 0.1)$. That is, because we cannot distinguish receiving fewer photons with higher coupling efficiency from receiving more photons with lower coupling efficiency, the correct choice of normalization lies in $\eta\in(0.99, 1)$ and we do not know what it is. The best we can do is to take the average required normalization across the detector. This results in a systematic error which depends on the scene being imaged, and which is most pronounced when the antenna factor changes rapidly over the width of the detector. Thus, with a wider detector width, this error increases.

We formalize our treatment of this error below. Consider a measurement made with a detection bin wider than the diffraction limit of the active metasurface. We start our calculation from an image, $N_{ideal}[k_{x}, k_{y}]$, where $k_{x}, k_{y}$ values are spaced within the diffraction limit. A non-diffraction-limited measurement $N_{detected, nd}\left[ \tilde{k_{x}}, \tilde{k_{y}} \right]$ gathers a photon count which can be approximated as

$$\begin{aligned} N_{detected, nd}\left[ \tilde{k_{x}}, \tilde{k_{y}} \right]=\sum_{k_{i}\in\tilde{k_{i}}\pm\Delta k_{d}} N_{detected}\left[ k_{x}, k_{y} \right]\#\left( S69 \right) \end{aligned}$$

Here, $\tilde{k_{x}}, \tilde{k_{y}}$ indicate the center of our non-diffraction limited measurements, which are more sparsely distributed than the diffraction-limited measurements. As before, we estimate our ideal photon count as

$$\begin{aligned} \tilde{N}_{ideal, nd}\left[ \tilde{k_{x}}, \tilde{k_{y}} \right]=\eta_{avg}^{-1}\left[ \tilde{k_{x}}, \tilde{k_{y}} \right]N_{detected, nd}\left[ \tilde{k_{x}}, \tilde{k_{y}} \right]\#\left( S70 \right) \end{aligned}$$

where $\eta_{avg}\left[ \tilde{k_{x}}, \tilde{k_{y}} \right]$ is an average efficiency expected across the measurement and accounts for a material efficiency (lowered by material absorption) and a coupling efficiency (which we average across all angles which contribute to the measurement)

$$\begin{aligned} \eta_{avg}\left[ \tilde{k_{x}}, \tilde{k_{y}} \right]=\eta_{material}\left[ \tilde{k_{x}}, \tilde{k_{y}} \right]\cdot{AVG}_{k_{i}\in\tilde{k_{i}}\pm\Delta k_{d}}(\eta_{coupling}\left[ k_{x}, k_{y} \right])\#\left( S71 \right) \end{aligned}$$

This efficiency $\eta_{avg}$ is our best guess as to the efficiency of a measurement. However, as explained, if the efficiency of the coupling varies significantly over the range of angles covered by a single measurement, it may not be exact. In contrast, we assume that $\eta_{coupling}[k_{x}, k_{y}]$ can be treated as constant over our diffraction-limited bin size. Then, our actual ideal photon count can be described as

$$N_{ideal, nd}\left[ \tilde{k_{x}}, \tilde{k_{y}} \right]=\sum_{k_{i}\in\tilde{k_{i}}\pm\Delta k_{d}} \eta^{-1}\left[ k_{x}, k_{y} \right]N_{detected}\left[ k_{x}, k_{y} \right]$$

Thus, we define a per-measurement normalization error which we assume is independent of shot noise

$$\begin{aligned} E\left[ \tilde{k_{x}}, \tilde{k_{y}} \right]=N_{ideal, nd}\left[ \tilde{k_{x}}, \tilde{k_{y}} \right] {- \tilde{N}}_{ideal, nd}\left[ \tilde{k_{x}}, \tilde{k_{y}} \right]\#\left( S72 \right) \end{aligned}$$

where the $N_{ideal, nd}$ corresponds to the true ideal collection count, without the normalization error. Then, we modify Eqn. S44 to include this error our SNR

$$\begin{aligned} SNR=\frac{\sum_{\tilde{k_{x}}, \tilde{k_{y}}} \tilde{N}_{ideal, nd}\left[ \tilde{k_{x}}, \tilde{k_{y}} \right]^{2}}{\sum_{\tilde{k_{x}}, \tilde{k_{y}}} \eta_{avg}^{-1}\left[ \tilde{k_{x}}, \tilde{k_{y}} \right]N_{ideal, nd}\left[ \tilde{k_{x}}, \tilde{k_{y}} \right]+E^{2}\left[ \tilde{k_{x}}, \tilde{k_{y}} \right]}\#\left( S73 \right) \end{aligned}$$

We use this equation to generate the SNR points in Fig. S6b. We find that for small bin sizes, the shot noise contribution to the error dominates the system—in this regime, the total acquisition time is important. However, if the bins are made too wide, the normalization error dominates the SNR of the system and acquisition time ceases to matter.

In Fig. 6a of the manuscript, we consider only the effects of shot noise on SNR, because we simulate image acquisition in the diffraction-limited regime.

**REFERENCES**

[1] C. A. Balanis, *Antenna theory: analysis and design*, 3rd ed. Hoboken, NJ: John Wiley, 2005.

[2] G. K. Shirmanesh, R. Sokhoyan, P. C. Wu, and H. A. Atwater, “Electro-optically Tunable Multifunctional Metasurfaces,” *ACS Nano*, vol. 14, no. 6, pp. 6912–6920, Jun. 2020, doi: 10.1021/acsnano.0c01269.

[3] MoSys, “Mosys | Bandwidth Engine 3 – BURST Memory IC.” Accessed: Mar. 03, 2025. [Online]. Available: http://mosys.com

[4] S. Kim, J. Kim, K. Kim, M. Jeong, and J. Rho, “Anti-aliased metasurfaces beyond the Nyquist limit,” *Nat Commun*, vol. 16, no. 1, p. 411, Jan. 2025, doi: 10.1038/s41467-024-55095-z.

[5] R. Sokhoyan *et al.*, “Electrically tunable conducting oxide metasurfaces for high power applications,” *Nanophotonics*, vol. 12, no. 2, pp. 239–253, Jan. 2023, doi: 10.1515/nanoph-2022-0594.

[6] R. Sokhoyan, C. U. Hail, M. Foley, M. Y. Grajower, and H. A. Atwater, “All-Dielectric High-Q Dynamically Tunable Transmissive Metasurfaces,” *Laser & Photonics Reviews*, vol. 18, no. 6, p. 2300980, 2024, doi: 10.1002/lpor.202300980.

[7] P. Thureja, G. K. Shirmanesh, K. T. Fountaine, R. Sokhoyan, M. Grajower, and H. A. Atwater, “Array-Level Inverse Design of Beam Steering Active Metasurfaces,” *ACS Nano*, vol. 14, no. 11, pp. 15042–15055, Nov. 2020, doi: 10.1021/acsnano.0c05026.

[8] R. C. Gonzalez and R. E. Woods, *Digital image processing*. New York, NY: Pearson, 2018.

[9] H. H. Barrett and K. J. Myers, *Foundations of Image Science*. John Wiley & Sons, Inc., 2004. Accessed: Sep. 12, 2023. [Online]. Available: https://www.wiley.com/en-us/Foundations+of+Image+Science-p-9780471153009

[10] R. Sun *et al.*, “Survey of Image Edge Detection,” *Front. Signal Process.*, vol. 2, Mar. 2022, doi: 10.3389/frsip.2022.826967.

[11] X. Ding, X. Zhang, J. Han, and G. Ding, “Scaling Up Your Kernels to 31×31: Revisiting Large Kernel Design in CNNs,” in *2022 IEEE/CVF Conference on Computer Vision and Pattern Recognition (CVPR)*, Jun. 2022, pp. 11953–11965. Accessed: Jun. 24, 2024. [Online]. Available: https://ieeexplore.ieee.org/document/9880273

[12] M. Davenport *et al.*, “The smashed filter for compressive classification and target recognition - art. no. 64980H,” *Proceedings of SPIE*, vol. 6498, Feb. 2007, doi: 10.1117/12.714460.

[13] L. López-García, W. Cruz-Santos, A. García-Arellano, P. Filio-Aguilar, J. A. Cisneros-Martínez, and R. Ramos-García, “Efficient ordering of the Hadamard basis for single pixel imaging,” *Opt. Express, OE*, vol. 30, no. 8, pp. 13714–13732, Apr. 2022, doi: 10.1364/OE.451656.
